# Bioluminescent Multi-Characteristic Opsin for Simultaneous Optical Stimulation and Continuous Monitoring of Cortical Activities

**DOI:** 10.3389/fncel.2021.750663

**Published:** 2021-10-25

**Authors:** Darryl Narcisse, Sourajit Mitra Mustafi, Michael Carlson, Subrata Batabyal, Sanghoon Kim, Weldon Wright, Samarendra Kumar Mohanty

**Affiliations:** Nanoscope Technologies LLC, Bedford, TX, United States

**Keywords:** Ca^2+^ bioluminescence multi-characteristic opsin (bMCOII), optogenetic, visual cortex, neural activity, neuromodulation, circadian rhythm

## Abstract

Stimulation and continuous monitoring of neural activities at cellular resolution are required for the understanding of the sensory processing of stimuli and development of effective neuromodulation therapies. We present bioluminescence multi-characteristic opsin (bMCOII), a hybrid optogenetic actuator, and a bioluminescence Ca^2+^ sensor for excitation-free, continuous monitoring of neural activities in the visual cortex, with high spatiotemporal resolution. An exceptionally low intensity (10 μW/mm^2^) of light could elicit neural activation that could be detected by Ca^2+^ bioluminescence imaging. An uninterrupted (>14 h) recording of visually evoked neural activities in the cortex of mice enabled the determination of strength of sensory activation. Furthermore, an artificial intelligence-based neural activation parameter transformed Ca^2+^ bioluminescence signals to network activity patterns. During continuous Ca^2+^-bioluminescence recordings, visual cortical activity peaked at the seventh to eighth hour of anesthesia, coinciding with circadian rhythm. For both direct optogenetic stimulation in cortical slices and visually evoked activities in the visual cortex, we observed secondary delayed Ca^2+^-bioluminescence responses, suggesting the involvement of neuron-astrocyte-neuron pathway. Our approach will enable the development of a modular and scalable interface system capable of serving a multiplicity of applications to modulate and monitor large-scale activities in the brain.

## Introduction

Understanding the activation paradigm of sensory processing requires stimulation and continuous monitoring of neural activities at cellular resolution. To associate the activities of a neural network with a specific condition, monitoring of neural activities at high spatiotemporal resolution is essential. Imaging and neuromodulation have become integral in the management of chronic pain and other neurological disorders such as Alzheimer’s disease, Parkinson’s disease, and depression (Brittain and Cagnan, 2018; Widge et al., 2018). However, a consensus has emerged in the neuromodulation field that there is a distinct need for the development of new methods for feedback-controlled modulation of neural activities on a circuit scale (Brain Initiative, 2019) for better control in pain management and therapies for movement disorders, epilepsy, etc. Although widely used in clinical settings, current methods for the modulation and detection of neural activities are based on electromagnetic fields (Peterchev et al., 2012), which have low spatial resolution for both stimulation and imaging (Luan et al., 2014) and lack cellular specificity. Cell-specific genetic targeting of neurons for optical stimulation and imaging with high spatial and temporal resolution (Pama et al., 2013) has emerged as a research tool and provides opportunities for clinical translation such as restoration of vision (Baker and Flannery, 2018). In order to restore vision that is lost because of an enucleated eye or optic nerve damage, current attempts based on electrical stimulation of primary visual cortex are limited by invasiveness, poor resolution (as higher electrode density requires more current, leading to heat production), and scar-tissue formation around implanted electrodes. Optogenetics has potential for non-contact, cell specific stimulation and recording, thus enabling bidirectional control over neural activities in visual cortex.

While numerous modifications of light-sensitive channels (opsins) have allowed the wavelength-selective precise modulation of cellular activities, optical recordings of neural activities are advancing at a rapid pace with genetically encoded fluorescence indicators (Lütcke et al., 2010; Chen et al., 2013; Marvin et al., 2013; Gong et al., 2015; Ji et al., 2016; Lin and Schnitzer, 2016; Weijian and Yuste, 2017). There is a greater variety and understanding of fluorescence indicators, and there is no requirement of a substrate that enables a simpler workflow for research applications. However, the requirement of excitation light for fluorescence may interfere with the schedule of photoactivation, unless a distinct spectral separation between excitation light and the photoactivation band is ensured. Furthermore, external excitation light induces phototoxicity (Benson et al., 1985; Laissu et al., 2017), which can hinder long-term observation of neural activities in living organisms. In addition, tissues are auto-fluorescent, leading to higher background noise for fluorescence-based measurements. Since bioluminescence does not require external excitation light, assays based on luciferases have been performed to image living organisms (Inouye et al., 2000). However, bioluminescence (Kotlobay et al., 2018) from these luciferases is several orders of magnitudes lower than fluorescence and, thus, will require the integration of signal that can hinder neural activity monitoring. In contrast, nano-lanterns (eNLs), generated by the fusion of a luminescent protein from oplophorus luciferase (derived from the deep-sea shrimp *Oplophorus gracilirostris*) with a fluorescent protein, have extremely bright luminescence (Hall et al., 2012; Takai et al., 2015) and do not require excitation light. Furthermore, the incorporation of Calmodulin-M13 domain into green nano-lanterns (GeNLs) produced Ca^2+^-sensitive bioluminescence with minimal light emission at basal Ca^2+^ levels. GeNL-Ca^2+^ has been employed to monitor pharmacologically evoked activities in dissociated cells (Suzuki et al., 2016).

Here, we describe the development and implementation of bioluminescent Multi-Characteristics Opsin (bMCOII), a hybrid optogenetic actuator and bioluminescence Ca^2+^-sensor, for excitation-free continuous monitoring of optically activated neural activities in cortical slices as well as in the visual cortex, with high spatiotemporal resolution that fundamentally extends the realm of neuromodulation and imaging. The combination of the highly photosensitive Multi-Characteristic Opsin (Wright et al., 2017) with the bright, BRET-based calcium indicator (Suzuki et al., 2016) allows for the simultaneous stimulation of transfected cells with a wide range of light wavelengths *via* the opsin, as well as monitoring of the activities of the cell *via* changes in GeNL-Ca^2+^ bioluminescence signal. This creates a tool that can report on internal cellular activities as a result of endogenous changes as well as induced changes from the cell-specific activation of the opsin. The uninterrupted recording of visually evoked neural activities in the cortex of mice enabled the determination of strength of sensory activation, which peaked at the seventh to eighth hour of anesthesia, coinciding with circadian rhythm (Paladino et al., 2013; Lezak et al., 2017). For better quantification of network activities, we also employed an artificial intelligence-based decoding algorithm, which transforms the time-lapse Ca^2+^ bioluminescence measurement peaks to a list of position coordinates and determines the communication pathway between them. Our approach will enable the development of a modular and scalable interface system capable of serving a multiplicity of applications to modulate and monitor large-scale activities in the brain.

## Materials and Methods

### Ethics Statement

All the experimental procedures were conducted according to the Institutional Animal Care and Use Committee (IACUC)-approved protocol of Nanoscope Technologies.

### Viral Packaging of Fusion Constructs of Multi-Characteristic Opsin and Bioluminescence Probe

The bMCOII plasmid is synthesized by the fusion of mutated opsins from algae (Chlamydomonas) and luminescent protein GeNL^20^. GeNL is a concatenation of a bright luciferase (NanoLuc, derived from the deep-sea shrimp *Oplophorus gracilirostris*) with green fluorescent protein (GFP). The N-terminus of the GeNL-Ca^2+^ gene was fused with the C-terminus of the MCOII gene, synthesized using a DNA synthesizer, and the sequence was verified. The synthesized GeNL-Ca^2+^-fused MCOII plasmid (bMCOII) was cloned into a pAAV MCS vector *via* its BamH1 and *Xho*I sites. We used the synthesized plasmid of bMCOII with a CAG promoter and packaged it into adeno-associated virus (AAV) 2/5 by triple transfection of RepCap, Helper, and bMCOII plasmids in human embryonic kidney 293 (HEK293)T cells. AAV2/5 physical titers were obtained by quantitative PCR using primers designed to selectively bind the AAV inverted terminal repeats. The concentration of virus used was 1.23 × 10^13^ VG/ml in order to ensure transfection over a wide area was achieved when directly placed on the cortical surface.

### Homology Modeling and Molecular Dynamics Simulation in Bioluminescence Multi-Characteristic Opsin

An initial 3D structural model from the amino acid sequence of bMCOII was obtained *via* homology modeling using Phyre2 (Kelley et al., 2015). Starting from this initial 3D structural model, structure refinement was performed with PREFMD (Feig and Mirjalili, 2016; Heo and Feig, 2018) and molecular dynamics (MD) simulation. In the final refined structure, channel openings were evaluated using BetaCavityWeb (Kim et al., 2015). The energy minimized docking of all-trans retinal (ATR) with the refined 3D structure of bMCOII was performed with the help of Autodock4 (Forli et al., 2016). At initial stages of calculation, the ATR molecule was made flexible, while the aromatic residues in the binding pocket of bMCOII were kept rigid. In subsequent steps of docking, both the ATR molecule and the binding pocket of protein were made flexible. A consistent solution was obtained after 20 rounds of iterations.

### Ca^2+^-Bioluminescence Imaging in Human Embryonic Kidney 293 Cells

The HEK293 cells were transfected using lipofectamine and the bMCOII plasmid when they became 70–80% confluent (after 5 days of culture in petri dish). For transfection, 2 μg of DNA and 4 μl of a Lipofectamine reagent in 2 ml of 10% Dulbecco’s Modified Eagle Medium (DMEM) was used. Three days after the transfection, Ca^2+^ bioluminescence recording was carried out using an EMCCD camera (Cascade1K; Photometrics, Tucson, AZ, United States) with a camera exposure time of 50 ms, which allowed for the recording of images at 20 Hz. One day prior to the recording, 10 μl of 100-μM ATR was added to the cells. The cells were stimulated using LED light pulse with duration of 100 ms, intensity of 15 μW/mm^2^. A control experiment was recorded without light illumination after the addition of an appropriate amount of substrate (25 μM furimazine, required for chemical reaction to provide energy for bioluminescence). To reduce ghosting artifact in the EMCCD camera resulting from exposure during light stimulation, deadtime calibration was achieved by the synchronization of stimulation pulse and camera recording. For the recording of high-resolution images in HEK cells, a confocal microscope with a reduced pinhole size of 125 μm along with 40 × oil immersion objective was used.

### Patch Clamp Recording Setup

The patch-clamp recording setup (Supplementary Figure 1A) consists of a micropipette (resistance: 20 MΩ) system that was filled with a solution containing (in mM) 130 K-gluconate, 7 KCl, 2 NaCl, 1 MgCl_2_,0.4 EGTA, 10 HEPES, 2 ATP-Mg,0.3 GTP-Tris, and 20 sucrose. The micropipette electrode was mounted on a motorized micromanipulator (MP225, Sutter Instruments, Novato, CA, United States). The extracellular solution containing (in mM): 150 NaCl, 10 Glucose, 5 KCl, 2CaCl_2_, 1MgCl_2_ was buffered with 10 mM HEPES (pH 7.3). The electrophysiological signals from the amplifier were digitized using Digidata 1440 (Molecular Devices, San Jose, CA, United States), interfaced with patch-clamp software (pClamp; Molecular Devices). For simultaneous Ca^2+^-bioluminescence measurement and photocurrent recording, Arduino UNO was used to synchronize the stimulation light pulses from the cool white LED (400–700 nm), EMCCD camera (Cascade1K; Photometrics) recording and photocurrent measurement with a MultiClamp system. The HEK293 cells were stimulated using LED light pulse with duration of 100 ms, intensity of 15 μW/mm^2^. The recording of Ca^2+^ bioluminescence was performed using the EMCCD camera with an exposure time of 50 ms. Ca^2+^ bioluminescence images were analyzed using ImageJ, and the photocurrent data from patch-clamp were processed using the pClamp10.7 software.

### Mouse Preparation

Wild-type (C57BL/6J) mice were obtained from Jackson Laboratory (Bar Harbor, ME, United States) and bred in the animal facilities of Nanoscope Technologies. The mice were maintained on a 12:12 (day: night) light cycle and treated humanely in strict compliance with IACUC on the use of animals in research.

### Immunoassay

In the immunostaining reaction, β3-tubulin and glial fibrillary acidic protein (GFAP) were used as markers for neurons and astrocytes, respectively. Freshly sectioned cortical slices from mouse visual cortices were made permeable by washing them in Triton X (0.5% of triton in 1X phosphate-buffered saline (PBS) solution). This was followed by overnight incubation at 4°C in a 4% goat serum blocking solution containing primary antibodies, 1:200 rabbit anti-GFAP, and 1:1,000 mouse anti-β3-tubulin. This was followed by subsequent washing in the Triton X washing solution. The slices were then immunostained with fluorescent tagged secondary antibodies goat anti-rabbit (Alexa 594, 1:250) and goat anti-mouse (Alexa 568, 1:250), for 1 h at room temperature. This was followed by three washing steps, again, using the Triton X washing solution. Lastly, 4’,6-diamidino-2-phenylindole (DAPI) staining was performed by adding 15 μM of DAPI solution in the sample for 15 min at room temperature. The additional DAPI was removed by washing three times in the 1X PBS solution. The immunostained tissue samples were kept in darkness for 2 h to dry and then mounted on a glass slide and cover slipped with mounting media for imaging. The images are taken in a confocal microscope using a 20 × oil immersion objective.

### Cranial Window Surgery

The mice were anesthetized with an intraperitoneal injection of ketamine to reduce swelling and inflammation. After the anesthesia reached appropriate depth, fur in the cranial area was removed chemically. A midline incision through the cranial skin provided access to the skull. The attached nuchal musculature was retracted, and the periosteum was scraped away. A 4-mm^2^ window was opened over the right visual cortex using a dental drill. The AAV2/5 virus (1.23 × 10^13^ GC/ml) was injected into four locations (separated by 2 mm) in the right visual cortex using a 33-gauge needle Hamilton syringe. The tip of the syringe was gently introduced into the cortical surface to avoid any bleeding. Additional virus was then dropped using a siliconized pipette tip over the cortical area. Five minutes after the application of the virus solution, 10 μl of antibiotic (ciprofloxacin) was applied over the cortical surface to prevent any infection. A polydimethylsiloxane (PDMS) window ∼100 μm thick was then laid over the surgical area, fixed with cyanoacrylate gel, and secured with dental cement. Extreme care was taken for the dental cement not to spread into the cortical window area. The area of the visual cortex was kept as clean and transparent as possible. After completion of surgery and window implantation, the mice were injected with 500 μl of saline injection *via* the intraperitoneal (IP) route to dilute the effect of anesthesia. The mice were kept in a recovery cage for approximately 2–3 h before being returned to cages separated from any other mouse to prevent potential damage to the optical window.

### Ca^2+^-Bioluminescence Imaging in Visual Cortical Slices

Approximately 4–6 weeks after the AAV2/5 injection, the mice were anesthetized by an intraperitoneal injection of mixture of 2.5 μl/g ketamine, 0.25 μl/g xylazine, and 0.125 μl/g acepromazine. The mice were then secured. The thoracic cavity was opened to gain access to the heart. A fresh N-methyl-D-glucamine (NMDG)-HEPES solution (at 4°C) was introduced to the cardiovascular system *via* a needle to the left ventricle, while the right atrium was severed to allow for the outflow of blood. The perfusion was continued until the cardiovascular outflow was clear and the heart ceased beating. As soon as this occurred, the animal was decapitated, and the brain was extracted carefully to avoid damage to the cortex. The cortex was then immediately embedded for axial sectioning in 2% Agarose. Axial cortical slices (100 μm thickness) were collected *via* compresstome in a 0–2°C cutting solution. This entire process was kept within 20 min of death. The cortical slices were immersed in a room temperature recording solution (93 mM NMDG, 2.5 mM KCl, 1.2 mM NaH_2_PO_4_, 30 mM NaHCO_3_, 20 mM HEPES, 25 mM glucose, 5 mM sodium ascorbate, 2 mM thiourea, 3 mM sodium pyruvate, 10 mM MgSO_4_, and.5 mM CaCl_2_) and imaged using an inverted fluorescence microscope (Discovery; Molecular Devices) with light stimulation (12.5–25 μW/mm^2^). The Ca^2+^ bioluminescence recording was performed using a CCD camera (CoolSNAP K4; Photometrics) with a camera exposure time of 200 ms (to allow for detection of bioluminescence with signal to noise), which allowed for the recording of images at ∼3 Hz. Simultaneous electrical recording was performed in a multi-electrode array (MEA) plate (MEA60-200-50-ITO; Qwane Biosciences, Switzerland) using a 32-channel Omniplex system (Plexon Inc.). The data were collected with camera exposure times of 50, 100, and 200 ms. All the experiments were recorded with a 25-μM concentration of furimazine (Nano-Glo Luciferase assay; Promega, Madison, WI, United States). Fluorescence imaging was performed in blue (465 nm) and green (505–540 nm) channels to check for area of bMCOII transduction so that it can be positioned with respect to the MEA electrodes.

### Longitudinal Ca^2+^-Imaging Assay in Anesthetized Mice

The mice were exposed to 0–12-h (8 AM to 8 PM CST) light and 12- to 24-h (8 PM to 8 AM CST) dark phases, and the recording was carried out from 4 ZT (Zeitgeber time) hour (i.e., 0 hr. recording) to 18 + ZT hour (>14 h recording). For the longitudinal Ca^2+^-imaging assay, at ∼4 ZT hour, the mice were anesthetized by a slow flow of isoflurane (∼1%) and oxygen (4%), as starting proportion, *via* a mask attached to the mouth of the mice. Before the start of the experiment, the cortical surgical area was washed with 100 μl of the 1X PBS (pH 7.4) solution. The washing PBS was removed with a sterile applicator, and 10 μl of 25 μM furimazine was directly added in the visual cortex and left for 10 min before visual stimulation began. The optical window of the mice was attached to the camera *via* a magnetic attraction between the guide and the cover of the camera to ensure any shifting was kept to absolute minimum and the same visualization area was always in focus. This was left in place for the entirety of the experiment session. The cool white LED light (400–700 nm) for visual stimulation was placed 5 mm from the eyes of the mice. During the experiment, the mice were monitored continuously for vital signals. The proportion of gas was adjusted in such a way that the animals remained anesthetized throughout the whole period. Continuous images were recorded in the EMCCD camera (Cascade1K; Photometrics) with a 25X/1.05 NA objective and 100 ms exposure time (with recording speed of up to 10 Hz) synchronized with the triggering of visual stimulation. In the course of the longitudinal experiment, the substrate was replenished by adding 10 μl of 25 μM furimazine every 3 h using a micropipette to introduce the substrate into one of the spaces between the lens and the window implant. This dilution of furimazine in sterile PBS was sufficient to keep the area moist and healthy. The addition of more substrate is necessary, as the signal for Nano-Glo^®^ Luciferase Assay Reagent (Promega) is known to decay >50% within 3 h. The addition of substrate was not associated with any changes in bioluminescence as the relative increase in bioluminescence is controlled by calcium concentration in the local area, not the presence of substrate (so long as enough is present at any given time). Sterile eye drops were applied regularly to keep the eyes moisturized. The images were processed and quantified in ImageJ and further analyzed in Origin 2018. The calculation of fractional fluorescence intensity relative to the surrounding background also eliminated any effect of substrate addition on the results. Various pulse width (10, 30 ms), intensity (10–20 μW/mm^2^), and frequency of light stimulation settings were used to collect data. The camera exposure time was also varied from 50 to 200 ms, and the recording was performed in dead time mode. The detailed experimental setup is shown in Supplementary Figure 6. For studies on inhibition of neuron-astrocyte communication, 100 mM of a stock solution of norepinephrine was made in DMSO, which was then diluted to 1 mM in the 1X PBS solution, and 50 μl of 1-mM norepinephrine was applied on to the visual cortex.

### Artificial Intelligence Analysis on Neural Networks

A convolution neural network (CNN) algorithm (Ciresan et al., 2012) was used to evaluate neuronal communication in the visually evoked cortical Ca^2+^-bioluminescence recording. The CNN algorithm consisted of four convolutions, four ReLU activation functions, four max-pooling, and two fully connected layers. To train the CNN algorithm, visually evoked GCaMP6 (A genetically encoded calcium indicator) fluorescence images in the mouse visual cortex (Yildirim et al., 2019) were used. These training parameters were then applied to identify network architecture, including node and communication pathways in the visually evoked Ca^2+^-bioluminescence recording (Supplementary Figure 10). To quantify neuronal activities, neuronal activation parameter (NAP) is defined as $\sum_{n}J_{n}\,{\left(\Delta \,I|I_{0}\right)}_{n}$, where ‘*n*’ is the number of nodes of signal communication between neurons, *J*_*n*_ is the number of connections associated with each node, and ΔI/I_0_ is the fractional change in Ca^2+^-bioluminescence signal.

### Statistical Analysis

The data are expressed as the average (Av.) ± standard deviation (SD). The data of experimental repetitions and individual mice were analyzed by two-tailed Student’s *t*-test. A *p*-value of <0.05 was deemed significant.

## Results

### Light Stimulated Activation of Bioluminescence Multi-Characteristic Opsin-Transfected Human Embryonic Kidney 293 Cells Shows ms-Dynamics

**FIGURE 1:**
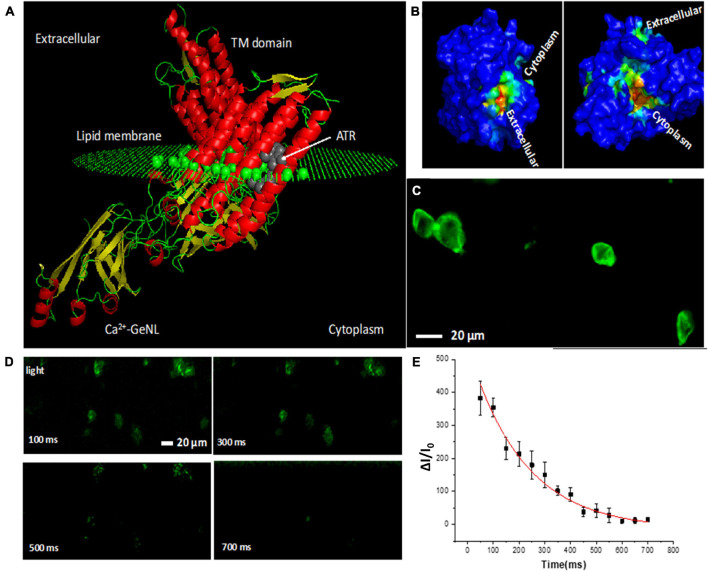
Modeling of bioluminescence multi-characteristic opsin (bMCOII) molecule, its expression, and functional characterization of Ca^2+^-bioluminescence in HEK293 cells upon light stimulation. **(A)** Three-dimensional structure of bMCOII in a lipid layer from homology modeling and molecular dynamics simulation along with bound ATR molecule (using Autodock 4). **(B)** Channel openings and cavity of the transmembrane bMCOII actuator domain as determined by simulation. **(C)** Confocal fluorescence image showing the expression of bMCOII in HEK293 cells. **(D)** Time-lapse change in Ca^2+^ bioluminescence (ΔI/I_0_, in%) from bMCOII-sensitized cells at various time points after light stimulation. **(E)** Temporal decay profile of Ca^2+^ bioluminescence signal with half-life of 216 ± 25 ms. *N* = 10 stimulations. Av. ± SD.

To understand the structure of the functional domains of bMCOII molecule, homology modeling and molecular dynamics (MD) simulation were carried out. Figure 1A shows the predicted 3D structure of bMCOII in the cellular lipid bilayer using Phyre2 and by MD simulation. The binding of the ATR molecule in the transmembrane (TM) domain was determined using Autodock 4. The hydrophobic core of the bMCOII-TM actuator domain constitutes the ATR binding site (Figure 1A), and the binding energy is calculated to be 8.4 Kcal/mole. The cation channel openings and cavity of the bMCOII-TM actuator domain were determined using BetaCavityWeb and are shown in Figure 1B. Upon stimulation with light, the opsin channel opens to allow for the passage of calcium into the transfected cells, which can then be immediately detected and reported by the Ca^2+^-GeNL sensor.

In order to correlate light stimulated Ca^2+^ bioluminescence activities with electrical activities, simultaneous optical and electrical recordings were carried out. The schematic of the optical stimulation integrated optophysiology (Ca^2+^ bioluminescence) and electrophysiology (patch clamp) setup is shown in Supplementary Figure 1A. Figure 1C shows the expression of bMCOII *via* a GFP-fluorescence reporter in the bioluminescence sensor, characterized by confocal fluorescence microscopy. The expression of bMCOII can be seen within the cells but is most intensely localized to the cell membrane. The bMCOII-transfected HEK293 cells showed minimal basal Ca^2+^-bioluminescence activities (without light stimulation) in the presence of furimazine. Upon stimulation with 100 ms pulse of light (15 μW/mm^2^), the cells transfected with bMCOII generated a threefold increase in Ca^2+^ bioluminescence intensity (Supplementary Figure 1B). The time-lapse Ca^2+^ bioluminescence mapping of cellular activities after light stimulation shows that the bMCOII- expressing cells are activated in a synchronized manner upon light stimulation (Figure 1D). Figure 1E shows the decay of Ca^2+^ bioluminescence to baseline with an off-time of 216 ± 15 ms. Supplementary Figure 1C shows a representative bioluminescence image 300 ms after light stimulation. While the light actuator domain of bMCOII is localized in the trans-membrane, the flanked Ca^2+^ bioluminescence sensor is designed to be in the intracellular space near the membrane. The light-dependent inward photocurrent in the bMCOII-expressed cells was measured with illuminating light pulses of 15 μW/mm^2^ intensity. A representative inward photocurrent profile from a bMCOII-transfected HEK293 cell shows a temporal response with an on-time (t_*on*_) of 32 ± 12 ms and an off-time of 68 ± 15 ms (Supplementary Figure 1D). In the bMCOII-sensitized cells, an inward photocurrent of 440 pA (in contrast to ∼200 pA recorded from MCO1 expressing HEK cells (21) was matched by a 230% increase in Ca^2+^ bioluminescence intensity. Concomitant plots of Ca^2+^ bioluminescence intensity and measured photocurrent show a significant correlation (Supplementary Figure 1E).

The fast rise and decay time of light-activated Ca^2+^ bioluminescence is attributed to the fusion of the GeNL-Ca^2+^ bioluminescence sensor and the MCO-II actuator. Having the calmodulin-M13 domain in the cytosolic side of the membrane, but just beneath the bMCOII-TM actuator domain (in the bMCOII-transfected cells) allowed for the binding of inward-flowing Ca^2+^ ions upon light-activation of the actuator. We compared the approach of co-expressing the same actuator (MCOII) and the Ca^2+^ bioluminescence sensor as separate proteins in the same cells. Supplementary Figure 2 shows that light stimulated bioluminescence activities in the HEK cells, expressing both the GeNL-Ca^2+^ bioluminescence sensor in cytosol and the MCO-II actuator in the membrane. Unlike the response of the bMCOII-transfected cells, the decay kinetics of Ca^2+^-bioluminescence in other cells in response to light stimulation (intensity: 20 μW/mm^2^) was found to be in the order of a few seconds (Supplementary Figure 2J). This is similar to what was reported in earlier literature (Suzuki et al., 2016).

### Enhancement of Ca^2+^ Bioluminescence Activities in Bioluminescence Multi-Characteristic Opsin-Sensitized Cortical Brain Slices Upon Increasing Light Stimulation Intensity

The visual cortex was transfected with AAV 2/5-carrying bMCOII 6–8 weeks prior to the recording. The tropism of adeno-associated virus (AAV2/5) (Castle et al., 2016) was utilized to enable the transduction of bMCOII in cortical neurons. Supplementary Figures 3A–C, respectively, show the fluorescence images of cortical slice immunostained with neuron-specific antibody (β3-tubulin), GFP-reporter of bMCOII, and nuclear stain (DAPI). The co-localization of β3-tubulin and bMCOII reporter fluorescence (Supplementary Figure 3D) confirmed the expression of bMCOII in cortical neurons. As shown in Supplementary Figure 3E, approximately 90% of the cells expressing bMCOII are cortical neurons. There was no detectable co-localization of bMCOII expression with GFAP antibody-stained astrocytes (Supplementary Figures 3F–I).

**FIGURE 2:**
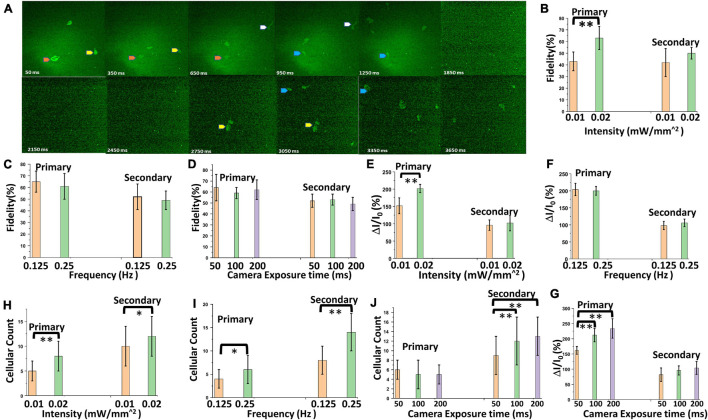
Characterization of Ca^2+^ bioluminescence in the bMCOII-sensitized visual cortex upon light stimulation. **(A)** Time-lapse images Ca^2+^ bioluminescence of a bMCOII-sensitized cortical slice stimulated with light (10 μW/mm^2^). **(B)** Fidelity of activation of selected neurons. **(C)** Fractional increase in Ca^2+^ bioluminescence intensity. **(D)** Number of active neurons, during primary and secondary response period as a function of light stimulation intensity. Variation in **(E)** fidelity. **(F)** Fractional increase in Ca^2+^ bioluminescence intensity. **(G)** Number of active neurons with the frequency of light stimulation pulses. **(H)** Fidelity of activation of selected neurons. **(I)** Fractional increase in Ca^2+^ bioluminescence intensity. **(J)** Number of active neurons as a function of camera exposure time. *N* = 70 neurons, 10 stimulations. Av. ± SD. *p < 0.05, **p < 0.01.

The optical stimulation and Ca^2+^ bioluminescence recording were conducted on bMCOII sensitized cortical brain slices in the presence of 25 μM furimazine. We measured the signal of Ca^2+^ bioluminescence at various camera exposure times (50–200 ms) and different light stimulation intensities (10–20 μW/mm^2^). Neural activities in the bMCOII-transfected cortical brain slices are segmented into two classes; those appearing within 500 ms of light stimulation were labeled as the primary response, while those appearing after 1 s (1,000 ms) were assigned as the secondary response (Figure 2A). This is based on our observation that the Ca^2+^ bioluminescence signal from an individual HEK cell (not receiving post synaptic stimulation) reaches the baseline value within 500 ms (Figure 1E). In the time interval of from 500 ms to 1 s (time sequence images from 500 to 950 ms in Figure 2A) after light stimulation, little or no Ca^2+^-bioluminescence activities were observed. For the primary response, significant enhancement in the fidelity of activation (reproducible signal from the same location of light-induced Ca^2+^ bioluminescence signal in all active neurons), activated fractional change in bioluminescence intensity (ΔI/I_0_, defined as the difference in the measured intensity of bioluminescent light divided by the ambient intensity measured before the visual stimulation was triggered), and number of active neurons was observed upon increasing the intensity of light stimulation, as shown in Figures 2B–D, respectively. However, the dependence of the secondary response on stimulation light intensity was found to be non-significant. In addition, the dependence of fidelity (Figure 2E) and bioluminescence intensity (Figure 2F) is found to be not statistically significant. On the other hand, increasing the frequency (repetition rate) of light stimulation enhanced active neuron count for both the primary and secondary responses (Figure 2G). Increasing camera exposure time did not improve the fidelity (Figure 2H), but improved signal integration and enhanced the signal-to-noise ratio, resulting in an increase of fractional change in Ca^2+^ bioluminescence intensity (Figure 2I) and cellular count of active neurons (Figure 2J) for primary and secondary responses.

### Spatiotemporal Correlation Between Ca^2+^ Bioluminescence Signal and Multi-Electrode Extracellular Potential in Bioluminescence Multi-Characteristic Opsin-Transduced Cortical Neurons Upon Light Stimulation

**FIGURE 3:**
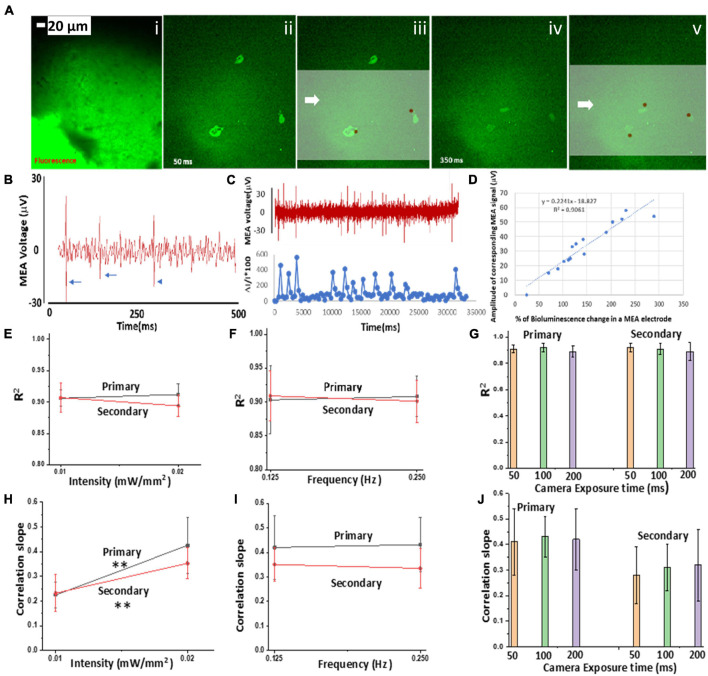
Correlation of Ca^2+^ bioluminescence and multi-electrode array (MEA) (MEA)-measured extracellular potential in bMCOII-sensitized cells upon light stimulation. **(A) (i)** Fluorescence image of a cortical slice on MEA; **(ii–v)** time-lapse images of Ca^2+^-bioluminescence signal: 50 **(ii–iii)** and 350 ms after **(iv–v)** stimulation with blue light at 470 ± 25 nm (intensity:0.5 mW/mm^2^), superimposed on the MEA measured extra-cellular potential (brown circles). Scale bar = 20 μm. **(B)** Representative voltage spikes (marked by arrows) recorded by one channel of the MEA. **(C)** Concomitant plot of MEA voltage (μV) spikes from a single MEA electrode and corresponding peaks of Ca^2+^-bioluminescence signal from a light-stimulated bMCOII-transduced neuron. **(D)** Correlation of% change in Ca^2+^ bioluminescence signal from neurons on the MEA electrodes with amplitude of corresponding voltage spike. R^2^: Coefficient of determination of the linear correlation plot between% change in Ca^2+^ bioluminescence signal and amplitude of corresponding voltage spike in MEA electrode. Dependence of R^2^ on **(E)** light stimulation intensity, **(F)** frequency of light stimulation, and **(G)** camera exposure time for the primary and secondary responses. Variation of slope of the linear correlation between% change in Ca^2+^ bioluminescence signal and corresponding voltage spike with **(H)** light stimulation intensity, **(I)** frequency of light stimulation pulses, and **(J)** camera exposure time. *N* = 3. Av. ± SD. **p < 0.01.

In order to correlate light-stimulated Ca^2+^ bioluminescence activities in the bMCOII-sensitized cortical neurons with electrical activities, simultaneous optical and electrical measurements were carried out. Figure 3Ai shows the fluorescence image of a cortical slice on a multi-electrode array (MEA). Extracellular voltage recordings of the bMCOII-transduced brain slices were performed using the MEA system at a sampling rate of 20 KHz. A bMCOII-transduced cortical slice was placed inside a temperature-controlled chamber over a transparent MEA plate, which was placed over an inverted fluorescence microscope. Synchronization between intracellular Ca^2+^-bioluminescence imaging, extracellular electrical recording, and light stimulation was carried out. The microscope field of view was centered and focused on the electrodes, and data acquisition was started prior to light stimulation, thus allowing for the monitoring of baseline activities. The representative time evolution of extracellular potentials after light stimulation (20 μW/mm^2^) in the MEA shows maximal activities (number of spikes and amplitude) at 500 ms, which drops down to minimum at 900 ms (Supplementary Figure 4). The overlay of MEA measured voltage spikes on Ca^2+^ bioluminescence images (Figure 3A, ii–iii) highlights the spatiotemporal correlation between them. Extracellular voltage spikes, upon light stimulation, from a bMCOII-transduced cortical slice recorded from an MEA electrode channel are shown in Figure 3B. The light stimulation of control cells (not transfected with bMCOII) did not evoke voltage spikes. A concomitant plot of change in Ca^2+^ bioluminescence signal and extracellular membrane potential for a single electrode shows a good correlation (Figure 3C). An analysis of the data from all 30 electrodes shows a significant correlation (R^2^ = 0.9) between% of Ca^2+^ bioluminescence intensity change and extracellular membrane potential (Figure 3D). The value of R^2^, the coefficient of determination of the linear correlation plot between% change in Ca^2+^ bioluminescence signal and amplitude of corresponding voltage spike, did not show a significant change for increase in stimulation light intensity (Figure 3E), frequency (Figure 3F), or exposure time (Figure 3G). The increase of light stimulation intensity was found to enhance the correlation slope between the two measurements for both the primary and secondary responses, which implies that intracellular Ca^2+^ bioluminescence intensity is approaching saturation at a stimulation intensity of 20 μW/mm^2^ and that the measured extracellular potential is within the dynamic range of the MEA sensors (Figure 3H). The correlation slope of two measurements was not found to have changed with change in light stimulation frequency (Figure 3I) or camera exposure time (Figure 3J).

In these experiments, using a marker (“11,” as seen in Supplementary Figure 5A, indicated by arrow) on the MEA, the spatial registration of neurons exhibiting light-induced Ca^2+^-bioluminescence, and MEA electrodes was achieved. MEA channels that recorded light-stimulated neuronal spikes are marked as black circles overlaid on the fluorescence image (Supplementary Figure 5B). Supplementary Figure 5C shows the time-integrated Ca^2+–^bioluminescence signal from the cortical slice observed upon stimulation with blue light (470 ± 25 nm). The registration of MEA channels activities with time-integrated Ca^2+–^Bioluminescence signal is shown in Supplementary Figure 5D (pseudo-color representing the order of appearance after light stimulation). The linear fit on the scatter plot between% of area around the electrode (diameter: 100 μm) showing Ca^2+–^bioluminescence activity and the total number of electrical spikes depicts a significant spatial correlation with R^2^ of.88 (Supplementary Figure 5E).

### Continuous *in vivo* Ca^2+^ Bioluminescence Monitoring of Neural Activities in the Visual Cortex Shows Enhanced Neural Activities Coinciding With Circadian Rhythm

To measure sensory activation strength during the circadian cycle, we carried out visual stimulation with varying stimulation parameters (light intensities: 13–22 μW/mm^2^; pulse widths: 10–30 ms) and continuous Ca^2+^ bioluminescence recording in the mouse visual cortex for >14 h. The mice were exposed to a 0–12-h light and 12–24-h dark phase, and recording was performed from 4 ZT (Zeitgeber time) (i.e., 0 h recording) to 18 + ZT h (>14 h recording). Supplementary Figure 6 (top) shows the schematic diagram of the *in vivo* setup used for the stimulation-synchronized recording of visually evoked Ca^2+–^bioluminescence cortical activities.

**FIGURE 4:**
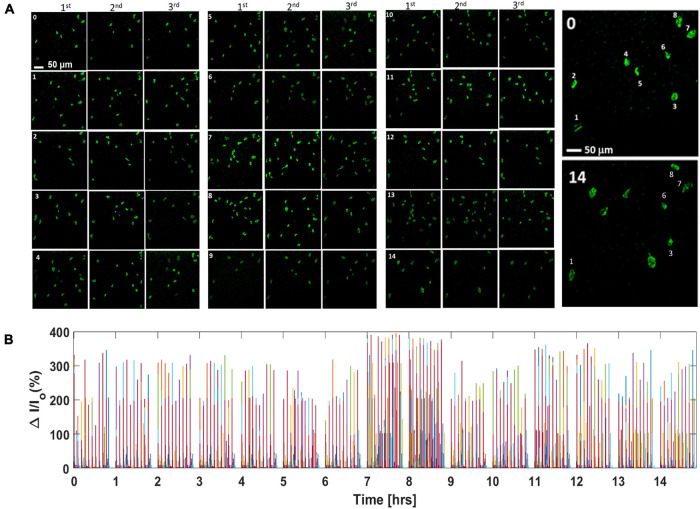
Continuous *in vivo* visually evoked Ca^2+^ bioluminescence activities in the bMCOII-transduced visual cortex of mice for extended duration. **(A)** Ca^2+^ bioluminescence signal after first three consecutive light stimulations at each time point (0–14 h; 0 = 0 h and 14 = 14th h). **(B)** Continuous plot of light stimulation-induced fractional increase in Ca^2+^ bioluminescence signal for the eight assigned active neurons for a 14-h period. Pulse width for light stimulation is 10 ms and intensity is 13 μW/mm^2^. Activities of different neurons are assigned with different colors.

**FIGURE 5:**
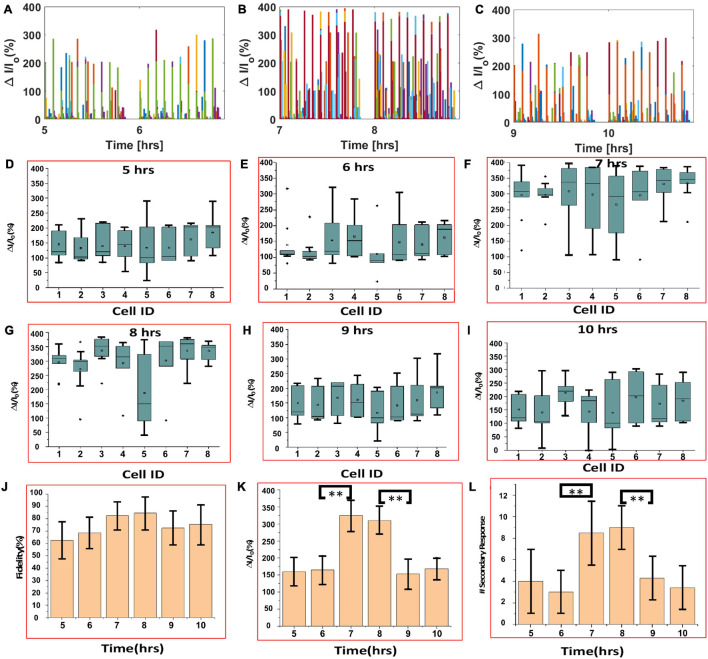
Analysis of continuous, extended *in vivo* visually evoked Ca^2+^-bioluminescence activities in the bMCOII-transduced visual cortex of mice. **(A–C)** Ca^2+^ bioluminescence changes for 5–6, 7–8, and 9–10 h, respectively. **(D–I)** Box plots showing average bioluminescence primary responses in the 5^*th*^, 7^*th*^, 9^*th*^, 6^*th*^, 8^*th*^, and 10^*th*^ hours, respectively, for each of the eight assigned neurons. **(J)** Fidelity, **(K)** fractional increase in primary bioluminescence signal, and **(L)** number of secondary responses from the eight assigned neurons. *N* = 10 stimulations. Av. ± SD. *p < 0.05 and **p < 0.01.

Calcium bioluminescence signals, immediately after the first three consecutive visual stimulations, at each time point, are shown in top panels of Figure 4. Repeatedly evoked Ca^2+^ bioluminescence signals in neurons of the visual cortex in the field of view were fast with consistently similar pattern. Supplementary Figure 7A shows the assignment of active neurons in the visual cortex at zeroth hour after the first visual stimulation, which could be identified at 14th hour imaging (Supplementary Figure 7B). In Figure 4B, we show a plot of light stimulation (pulse width: 10 ms and intensity: 13 μW/mm^2^)-induced fractional increase in Ca^2+^ bioluminescence signal for eight assigned active neurons for a 14-h period. Fractional enhancement of Ca^2+–^bioluminescence intensity from cortical neurons of the bMCOII-transduced visual cortex for 10 consecutive visual stimulations is shown in Supplementary Figure 7C. Similar to the direct light stimulation of bMCOII-transduced cortical slices (Figures 2, 3), a primary response, followed by a delayed and weaker secondary response, was observed. Periods of heightened Ca^2+^-bioluminescence activities were observed at 7–8 h after anesthesia (Figure 4), which were confirmed in multiple animals (Supplementary Figure 6 Bottom). Figure 4B, as well as the zoomed plots (Figures 5A–C), shows the enhancement of Ca^2+^ bioluminescence intensity from the assigned active neurons at 7–8 h for 14 h of recording. Box plots (Figures 5D–I) show the average bioluminescence (primary responses) intensity and variations across 10 stimulations in the 5th to 10th hour for each of the eight assigned neurons. The mean values for each of the assigned active neurons at 7–8 h were found to be higher than those in the preceding (i.e., fifth to sixth hour) or subsequent (i.e., 9th to 10th hour) recording in the anesthetized mice. The fidelity of sensory response to visual stimulation was >60% at all time points during the extended study (Figure 5J). Fractional change in Ca^2+^-bioluminescence activities (Figure 5K) and number of secondary responses from the eight assigned neurons (Figure 5L) were found to be significantly higher during the seventh to eighth hour. Supplementary Figure 7D shows decreased secondary response at 14th h of recording.

### Effect of Visual Stimulation Parameters on Circadian Rhythm-Dependent Ca^2+^-Bioluminescence Visual Cortex Activities

**FIGURE 6:**
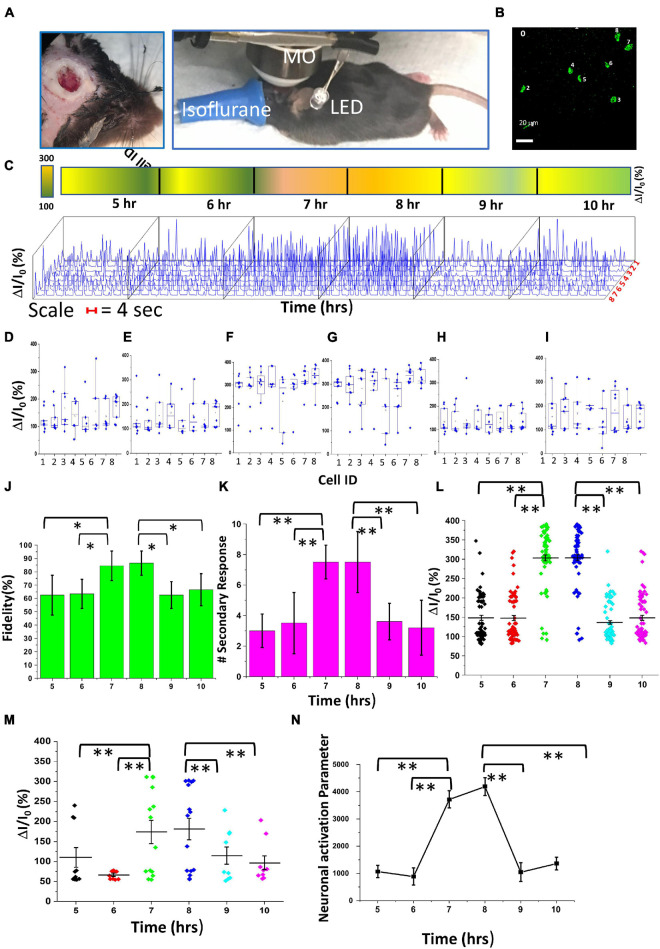
Comparison of *in vivo* Ca^2+^ bioluminescence activities and neural activation parameter in the visual cortex expressing bMCOII upon light stimulation at 5–10 h of recording. **(A)** Experimental setup for long-time recording of Ca^2+^ bioluminescence signal. MO: microscope objective (25×, NA = 1.05). **(B)** Ca^2+^ bioluminescence image after 1st light stimulation at 0th hour showing the eight assigned neurons. **(C)** 3D plot of fractional change in Ca^2+^ bioluminescence intensity for 10 stimulations at hours 5–10 during the experiment. **(D–I)** Box plots showing fractional change in Ca^2+^ bioluminescence intensity at 5–10 h interval. **(J)** Fidelity. **(K)** Number of secondary responses. Scatter plot of fractional change in bioluminescence signal for **(L)** primary response and **(M)** secondary response, for various stimulation parameters. **(N)** Calculated neuronal activation parameter (NAP) at 5–10 h interval. *N* = 10 stimulations. Av. ± SD. **p < 0.01; *p < 0.05.

To evaluate the effect of visual stimulation parameters on neural activities of the visual cortex, we varied the intensity and pulse width of the LED light while keeping the animals anesthetized over a >14-h period, and imaging was performed with a 25 × microscope objective (Figure 6A). The Ca^2+^-bioluminescence image at 0 h, following the first light stimulation, is taken as a reference, and neurons showing Ca^2+^bioluminescence response were assigned with numbers (Figure 6B); these numbered neurons were assigned as regions of interest for subsequent analysis. Changes in intensity within each region were measured as belonging to the neurons assigned with numbers. Supplementary Figure 8 shows the extended recording of Ca^2+–^bioluminescence signal in the bMCOII-transduced visual cortex upon low intensity (13 μW/mm^2^) visual stimulation (pulse width 30 ms). Continuous plot of fractional increase in Ca^2+^ bioluminescence signal for the assigned neurons activated by visual stimulation for the 14-h period is shown in Supplementary Figure 8A. Supplementary Figures 8B–D show maximal change in Ca^2+^ bioluminescence during the seventh to eighth hour of anesthesia as compared with that during the 5th–6th or 9th–10th-h period similar to 10 ms stimulation results (Figures 4, 5). The box plots in Supplementary Figures 8E–J show average bioluminescence responses in the 5th, 7th, 9th, 6th, 8th, and 10th hours, respectively, for each of the assigned neurons. Like the 10-ms stimulation (Figures 4, 5), there was no change in fidelity, over the 5- to 10-h period (Supplementary Figure 8K). However, the fractional increase in bioluminescence signal (Supplementary Figure 8L) and the number of secondary responses (Supplementary Figure 8M), due to 30-ms visual stimulation over the 7- to 8-h period, was higher. For higher visual stimulation light intensity, the visual cortex activities (measured by bioluminescence intensity profile of assigned peaks) over the 0- to 14-h period showed a similar trend in neural activation (Supplementary Figure 9). In addition, increasing the visual stimulation light intensity to 22 μW/mm^2^ (keeping the pulse width at 30 ms) led to a significant increase in the fidelity of activation during seventh to eighth hour (Supplementary Figure 9K), unlike low-intensity visual stimulation (Figure 6J and Supplementary Figure 8K).

A rigorous analysis of neural activities in the visual cortex was performed with 3D representation of the intensity profiles for the eight assigned neurons (Figure 6B) within the 5th–10th-h interval (Figure 6C). The percentage (%) increase in primary Ca^2+^ bioluminescence signals from the assigned active neurons showed a statistically significant enhancement during the 7th–8th hour of recording with visual stimulation intensity of 22 μW/mm^2^ and pulse width of 10 ms. Figures 6D–I respectively show the box plots of the average bioluminescence intensity and the variations across 10 stimulations in the 5th to 10th hours for each of the eight assigned neurons. Statistically significant increases in the fidelity of activation (Figure 6J), number of secondary responses (Figure 6K), percentage (%) Ca^2+^ bioluminescence primary signals (Figure 6L), and secondary signals (Figure 6M) during the 7- to 8-h period were observed.

Furthermore, we analyzed the neural activity patterns using the AI-based algorithm, which identifies the communication pathway between active neurons. The measure of neuronal network activation depends not only on the increase in Ca^2+^ bioluminescence intensity but also on the number of nodes and the number of connections associated with each node. Here, we introduce a new measure, neuronal activation parameter (NAP), defined as $\sum_{n}J_{n}\,{\left(\Delta \,I|I_{0}\right)}_{n}$, where “*n*” is number of nodes of signal communication between neurons, *J*_*n*_ is the number of connections associated with each node, and ΔI/I_0_ is the fractional change in Ca^2+^ bioluminescence signal. Supplementary Figure 10 shows the visually stimulated time-lapse bioluminescence mappings in the visual cortex and AI-assisted neural activity architecture (with nodes and pathways). Signal transmission traces for every light stimulation were superimposed (Supplementary Figure 10), where each node represents neurons that have at least three-way connections in the signaling pathway. The calculated visual cortex-NAP values in the 5- to 10-h period of visually stimulated neural activation are shown in Figure 6N. A fourfold increase in NAP-values can be seen during the 7- to 8-h period of anesthesia. Furthermore, to quantify the effect of pulse width and intensity of visual stimulation on the activation of network activities in the visual cortex, we compared fidelity, number of secondary responses, percentage (%) of Ca^2+^ bioluminescence intensity enhancement in the primary and secondary responses, and NAP at eighth hour of anesthesia (Supplementary Figure 11). With increased stimulation intensity, from 13 to 22 μW/mm^2^ (30 ms pulse width), the fidelity of activation (Supplementary Figure 11F) and NAP (Supplementary Figure 11J) showed a statistically significant enhancement.

We further monitored baseline Ca^2+^-bioluminescence activities in the bMCOII-transduced visual cortices of the mice without any light stimulation. This activity is likely due to a random or unrelated neural activity in the brain and may account for the isolated extra active neurons seen in some frames. The baseline neural activities were sparse, as shown in time-course Ca^2+^ bioluminescence signals from the visual cortex (Supplementary Figure 12A). The time integration of Ca^2+^ bioluminescence signals shows only a few active neurons (Supplementary Figure 12B). The time-integrated Ca^2+^ bioluminescence signal at each time point for a 0–14 h duration shows no apparent intensity enhancement (Supplementary Figure 12C) during the 7- to 8-h period of recording. Only a 50% fractional increase in Ca^2+^ bioluminescence signal relative to the background noise level was observed in active neurons for the 14-h period (Supplementary Figures 12D,E), which is much lower than the∼300% enhancement of signal seen in primary visually evoked responses. The time-averaged histogram profile of Ca^2+^ bioluminescence intensities is uniform without any significant signal enhancement for the 0- to 14-h period (Supplementary Figure 12F). A marginal enhancement in the total number of active neurons during the 6th-h period of recording (10th ZT h) was observed (Supplementary Figure 12G).

## Discussion

### Temporal Resolution and Sensitivity of Bioluminescence Multi-Characteristic Opsin: Actuator and Ca^2+^ Bioluminescence Sensor

At a light intensity of 15 μW/mm^2^, the temporal ON response of the bMCOII actuator was measured with patch clamp to be 32 ± 12 ms and that of the Ca^2+^ bioluminescence sensor was <50 ms (limited by imaging). The ON-time of the actuator is expected to be lower at higher stimulation intensity as reported for other opsins. The OFF-time of the sensor was measured to be 216 ± 25 ms in the HEK293 cells (Figure 1E) as compared with the OFF-time of 68 ± 15 ms for the bMCOII actuator. There may be several factors governing the off response of the Ca^2+^ bioluminescence sensor in bMCOII: (i) the response time of inherent Ca^2+^ sensor in bMCOII and (ii) structural perturbation that transmembrane helices of bMCOII induce upon the Ca^2+^ bioluminescence sensor. The transmembrane actuator has high affinity toward the cell membrane. However, if there is a structural interaction between transmembrane helices and Ca^2+^-sensory domain, it can perturb the overall temporal response. The 3D structure of bMCOII, as evaluated by homology modeling and MD simulation (Figure 1A), reveals that the ATR-bound TM-actuator domain of bMCOII is in the cell membrane and that the Ca^2+^-bound sensor flanks by a flexible inter-domain linker inside the cytoplasm near the cell membrane (∼20 Å). The fluorescence imaging by confocal microscopy (Figure 1C) supports the prediction obtained from MD stimulation and homology modeling.

GCaMP-based Ca^2+^-fluorescence signaling has been used extensively for monitoring Ca^2+^ activities both *in vitro* and *in vivo.* The binding affinity varies from.1 to 10 μM, and the ON-time and OFF-time ranges between 10–20 and 80–500 ms, respectively (Marco et al., 2006; Lin et al., 2012; Helassa et al., 2015; Podor et al., 2015; Zhijie et al., 2017). Recently, a GcaMP7 variant demonstrated a much faster off-time of around 20 ms. However, for neural activities leading to an extracellular potential of 40–60 μV (recorded by MEA), the change in GcaMP fluorescence-based system is typically within 150% with respect to baseline (Podor et al., 2015). In contrast, the Ca^2+^bioluminescence indicator in bMCOII shows a change of ∼300% corresponding to the 50-μV change in extracellular potential (Figures 3C,D). Furthermore, the bioluminescence-based system (bMCOII) does not need excitation light and, therefore, is not prone to photobleaching, thus allowing for the long-term monitoring of neural activities. The *in vitro* patch-clamp measurements show that direct stimulation of bMCOII expressing cells with a light intensity of 15 μW/mm^2^ generates a ∼ 400 pA inward current. This is because of the large funnel structure of the ion-conducting pore (diameter: outer ∼15 ^0^A, inner ∼7 ^0^A) of the light-activated ATR-bound bMCOII actuator. The highly sensitive nature of the bMCOII actuator allows for low power burden as well as no photochemical or photothermal damage in the neural tissue. The concomitant bioluminescence measurements showed that a 2-pA change in inward current (i.e., ∼0.1 mV change in membrane potential) leads to a ∼1-% change in Ca^2+^ bioluminescence signal. Previous measurements on the Ca^2+^ bioluminescence response of the GeNL-Ca^2+^ sensor in cardiac myocytes showed an OFF-time of ∼1 s. It may be noted that the pharmaceutically induced activity pattern (membrane depolarization and inward Ca^2+^ flow) of cardiomyocytes is slower than the Ca^2+^-sensor itself. However, the decrease in the observed response time in the bMCOII sensor may be primarily dictated by the structural perturbations in the sensor induced by the light-activated bMCOII actuator, which is connected to the sensor *via* a flexible inter-domain linker.

### Primary and Secondary Light Evoked Responses in Cortical Slices and Visual Cortex

Primary activity responses from neurons in the cortical slices expressing bMCOII after low intensity light (∼10 μW/mm^2^) stimulation were found to have high fidelity. The observation of delayed secondary response after the direct stimulation of bMCOII-transduced neurons in the cortical slices (Figures 2, 3) is intriguing. This was evident not only in Ca^2+^ bioluminescence response but also in the MEA response to a single 100-ms light stimulation pulse (Figure 3C). Since the AAV2/5 selectively transduces cortical neurons (Lezak et al., 2017), the secondary response is also from the neurons. The origin of the secondary Ca^2+^ bioluminescence response may be attributed to the signal transduction from the primary activities in light-activated neurons that are transmitted *via* astrocyte-neural coupling. The astrocyte-mediated glutamate pathway has been shown (Podor et al., 2015) to govern the signal transmission between neurons and astrocytes, and the neuro-inhibitor norepinephrine has been shown to disrupt this communication. Furthermore, the delayed (∼1 s after light stimulation) appearance of the observed secondary response may be attributed to the fact that Ca^2+^-response in astrocytes is much slower (∼100 ms) in contrast to signal transmission between neurons (∼ few milliseconds). In addition to direct stimulation in cortical slices, the secondary response was also observed in the bMCOII-transduced visual cortex (Supplementary Figures 7–11 and Figures 4, 5) upon visual stimulation at various intensities and pulse widths. Upon increasing light stimulation intensity, the activation fidelity (Figure 2B) and fractional change in primary Ca^2+^ bioluminescence response (Figure 2C) are enhanced in the bMCOII-transduced brain slices. Higher light stimulation intensity activates more bMCOII-actuator molecules to the increase passage of Ca^2+^ ions, leading to an increase in primary Ca^2+^ bioluminescence response. Since the delayed secondary response is hypothesized to be *via* the neuron-astrocyte-neuron pathway, the fractional changes in Ca^2+^ activities were found to be less affected by increasing intensity and frequency (Figures 2C,F). However, a significantly higher active neuron count was observed because of increased stimulation intensity in both the primary and secondary responses (Figure 2D). More interestingly, with increased frequency of light stimulation, the increase in the number of active neurons was higher for the secondary response than for the primary response (Figure 2G). This can be attributed to the recruitment of more neurons (including the primary response neurons) in the light-stimulated neural circuitry *via* the astrocyte-neuron pathway.

Furthermore, to confirm our hypothesis, we characterized visual stimulation-induced Ca^2+^ bioluminescence activities in the visual cortex by adding norepinephrine; an inhibitor of neural-astrocyte coupling (Yasmin et al., 2018). Upon light stimulation, both the primary (early, <0.5 s) and secondary (late, >1 s) responses were observed in the visual cortex (Supplementary Figure 13A). However, upon the addition of 1 mM norepinephrine, the secondary responses were completely inhibited (Supplementary Figure 13B). Plots of fractional change in Ca^2+^-bioluminescence response show the presence of strong secondary responses without norepinephrine (Supplementary Figure 13C), which completely disappears upon the addition of 1 mM inhibitor (Supplementary Figure 13D).

Strong temporal (Figures 3C,D) and spatial (Supplementary Figure 4) correlations of light-stimulated Ca^2+^ bioluminescence responses with extracellular potential were observed in the bMCOII-transduced brain slices. As shown in the immunohistochemistry experimental results (Supplementary Figure 3B), the bMCOII protein is largely membrane-bound and localized to the soma. Therefore, it is expected to have a bioluminescence signal emitted from the soma. Furthermore, since bioluminescence signals are weak (by nature), the Ca^2+^ bioluminescence signal from soma is better detected by the camera than that from the neurites (if any). In our analysis, we expected that any neuron firing within 50 μm from an MEA electrode would contribute to the observed voltage change. However, the dependence is not straightforward, since electrostatic potential varies as inverse of distance. Therefore, a more accurate correlation can be established by taking into account the weightage factor based on the exact distance between origin of bioluminescence signal (location of cell) and MEA. The slope between extracellular potential recorded by MEA electrode and% change in Ca^2+^ response in the bMCOII-transduced brain slices increased significantly with increase in light intensity for both the primary and secondary responses (Figure 3H). Upon light stimulation, the Ca^2+^ ions flow through the activated bMCOII-actuator domain and get bound to the bMCOII-sensor domain. When most of the sensor domains are bound to Ca^2+^, bioluminescence intensity will tend to reach saturation. If the ions continue to flow through the channel, it will result in an increase in extracellular potential. The dynamic range of the MEA electrical sensor is an order of magnitude higher than that of the Ca^2+^ bioluminescence sensor and, therefore, could better detect electrical activities at higher light intensities.

### *In vivo* Visually Evoked Cortical Ca^2+^ Bioluminescence Recording and Correlation With Circadian Rhythm

The mice were exposed to a 0- to 12-h light and 12- to 24-h dark phase, and the recording was done from 4 ZT (Zeitgeber time) h (i.e., 0 h recording) to 18 + ZT h (>14 h recording). Extended monitoring of visually evoked Ca^2+^ bioluminescence activities in the bMCOII-transduced visual cortex of mice showed peak activities at the seventh to eighth hour of anesthesia (Figures 4, 5 and Supplementary Figures 8, 9). To avoid depletion of the bioluminescence substrate (furimazine), we added a substrate every 3 h. In the absence of the substrate, no bioluminescence of any kind was observed. The addition of substrate with this selected interval did not change the basal or activated intensity level during the extended monitoring. We hypothesize that this is because the baseline enzymatic activity of GeNL-Ca^2+^ luciferase on furimazine is not affected by the local concentration of furimazine so long as a baseline threshold amount is available; it is instead limited by the availability of intracellular calcium ions. Moreover, the relative intensity of the induced change in bioluminescence is likewise regulated solely by the concentration of local calcium. As such, so long as the relative amount of calcium was similar because of the opening of the channel of the opsin or calcium changes secondary to depolarization of the neuron, there would be a similar bioluminescence signal evoked at any given time. Thus, the replenishment of furimazine merely kept the level of the locally available substrate above the minimum concentration without affecting the measurements. Furthermore, there was no correlation of observed fractional change in Ca^2+^ bioluminescence and related analysis (e.g., fidelity, active neurons) on stimulation (pulse width, intensity) and imaging (camera exposure time) parameter to the timing of substrate addition (Figures 4, 5 and Supplementary Figures 8, 9). The fractional change in Ca^2+^ bioluminescence is observed to be significantly high for both the primary and secondary responses (Figures 5L,M) during the 7th–8th recording hour (i.e., 11–12th ZT h). Furthermore, the fidelity in visually evoked primary and secondary responses in the visual cortex was significantly high during this period. In addition, during the 11th–12th ZT hour, Ca^2+^bioluminescence imaging showed that the total number of active neurons eliciting a secondary response reached a peak.

Enhancement in the intensity of Ca^2+^ bioluminescence response is a measure of neuronal signal strength, but it cannot quantify the neuronal activation of the underlying circuitry alone. For example, isolated neurons may have high signal enhancement, but may not have in an actively communicating network. On the other hand, a highly connected network might have weak signal strength. A highly connected network having a large number of nodes with strong signal strength is a measure of a neuronal network that can be highly activated. Therefore, CNN-based artificial intelligence was utilized to determine the structure of nodes (nodes and communication pathways) in the visually activated neuronal circuitry of the visual cortex during the extended recording period (0 to >14 h). At seventh to eighth recording duration, a maximum node structure appeared along with increased Ca^2+^ bioluminescence. To better characterize the network activity upon visual stimulation, neuronal activation parameter (NAP) was defined as a function of the number of nodes, the number of paths it connects, and the strength of Ca^2+^ bioluminescence signal, although under anesthesia, a fourfold increase in NAP in the interval of the seventh to eighth recording hour (Figure 6N) is consistent with the earlier observation of increased activities during this period in non-anesthetized mice (Paladino et al., 2013; Lezak et al., 2017). Continuous EEG monitoring of the for 24 h (12-h light and 12-h dark) shows basal level of activity counts at the 1st–9th ZT hours, which increases significantly (∼5X) at the 11th–12th ZT hour and reaches minimum at ∼18th –19th ZT hour (Paladino et al., 2013; Lezak et al., 2017). Furthermore, our observation of decrease in Ca2 + bioluminescence activities after the 7th–8th until > 14th hour of recording matches well with the reported trend (Paladino et al., 2013; Lezak et al., 2017). The autonomic response of the body due to circadian clock makes cortical neurons hyperactive with respect to visual stimulation, which is displayed in our extended Ca^2+^ bioluminescence imaging.

Bioluminescence, so far, has been reported for mesoscopic imaging *in vivo* with low resolution. To achieve cellular resolution for bioluminescence-based activity imaging in a 3D tissue environment, there have been significant ongoing efforts such as development of source localization algorithm and/or optical engineering. In this study, bioluminescence activity imaging at single cell resolution was achieved by designing a Ca^2+^ bioluminescence sensor that provides high signal-to-noise ratio with significantly improved temporal response to allow the use of a high numerical aperture imaging lens that rejects out-of-focus Ca^2+^ bioluminescence signal. Therefore, even in the 3D *in vivo* tissue environment, distinct Ca^2+^-bioluminescence activities at cellular resolution could be observed. Since the *in vivo* Ca^2+^-bioluminescence signal in the brain was observed in distinct cells after light stimulation of the eye, there was no scope of excitation light leakage or autofluorescence as encountered in fluorescence-based activity reports.

Time of day-dependent environmental changes modulates the circadian rhythm, which is known to affect physiology. While in animal and humans such changes have been characterized by protein dynamics, electrophysiology, and fMRI studies, no report exists on single-cell resolution activity imaging to demonstrate time-of–day-variation of visual perception *in vivo* in rodents. We used Ca^2+^ bioluminescence to study brain dynamics during resting-state and close-to-threshold visual activation repeatedly over >14 h. The visual perception (at resting-state and close-to-threshold visual activation) was found to be remarkably enhanced at 11th–12th h ZT time. Disruption of cellular activities can also happen because of different common environmental factors such as stress, dietary habits, and addiction, and there is a need for high-resolution and long-term imaging methods such as bioluminescence, as presented here, to better understand the functioning and changes in brain activities and associated cognitive processes. Although the data presented here demonstrate the excitation-free, long-term, and continuous monitoring of visual cortex activities at single cell resolution with a high signal-to-noise ratio, Ca^2+^ bioluminescence monitoring is directly applicable to many fields of neuroscience including vision, other sensory responses, as well as for longitudinal studies on cellular activities of other organs (e.g., the heart), and their modulation due to sleep, disease state, and therapeutic interventions.

Furthermore, we demonstrated the MCO-based modulation of cortical neural activities with very low power of light. We envision using MCO for lowering the power burden for stimulation of the visual cortex and other cortical areas to restore vision in subjects with enucleated eye or optic nerve damage or suffering from chronic pain and other neurological disorders.

## Conclusion

The hybrid optogenetic actuator and Ca^2+^-bioluminescence sensor (bMCOII) enabled the simultaneous modulation and excitation-free imaging of neural activities. The bMCOII actuator was activated at very low intensities with fast on- and off-responses, and the activated neurons were simultaneously detected by a highly sensitive Ca^2+^ sensor with an enhanced spatial and temporal resolution. The observed secondary neural activity response after direct light stimulation was also exhibited during indirect (visual) stimulation, as measured by Ca^2+^ bioluminescence correlated with electrical measurements. The excitation-free imaging allowed continuous monitoring of visual cortical activities over a >14-h period without photobleaching or tissue damage. Detailed characterization of the light-activated Ca^2+^-bioluminescence activities (i.e. Intensity change, fidelity and number of secondary responses at different light intensities, frequencies of stimulation, pulse widths times) showed peak visual cortical activities, in consistence with circadian rhythm. The artificial intelligence-based decoding algorithm allowed the defining of NAP based on Ca^2+^ bioluminescence activities, nodes, and communication pathways for better interpretation of the network activities. The use of bMCOII for low-power modulation and simultaneous quantification of network activity patterns by excitation- and substrate-free bioluminescence (Kotlobay et al., 2018) imaging will enable the development of a modular and scalable interface system for bidirectional control capable of serving a multiplicity of applications in neuroscience and medicine.

## Data Availability Statement

The original contributions presented in the study are included in the article/Supplementary Material, further inquiries can be directed to the corresponding author/s.

## Ethics Statement

The animal study was reviewed and approved by Nanoscope IACUC.

## Author Contributions

DN, SMM, MC, and SKM carried out stimulation, imaging, and electrical recording experiments on cortical slices. SMM carried out molecular dynamics simulation and homology modeling of bMCOII, performed AI analysis, and supervised the project. SB and SMM carried out experiments on HEK cells and patch clamp electrophysiology. DN carried out cortical injections and optical window implant. DN, SMM, and SKM conducted *in vivo* imaging. DN and SMM carried out immunohistochemistry analysis. SK performed data analysis and made graphical presentations. All authors participated in discussion and data analysis and contributed to the preparation of the manuscript.

## Conflict of Interest

All authors were employed by the company Nanoscope Technologies LLC. SKM has equity interest in Nanoscope Technologies, LLC, which is developing products in biomedical diagnostics and therapeutic technologies.

## Publisher’s Note

All claims expressed in this article are solely those of the authors and do not necessarily represent those of their affiliated organizations, or those of the publisher, the editors and the reviewers. Any product that may be evaluated in this article, or claim that may be made by its manufacturer, is not guaranteed or endorsed by the publisher.
