# Supplementary material for: Bioluminescent Multi-Characteristic Opsin for Simultaneous Optical Stimulation and Continuous Monitoring of Cortical Activities
Source: Front Cell Neurosci. 2021 Oct 25;15:750663. doi: 10.3389/fncel.2021.750663 (PMC8573050; doi:10.3389/fncel.2021.750663)
Supplement: Supplementary file 1 [file Data_Sheet_1.pdf]

Supplementary Material

Bioluminescent Multi-Characteristic Opsin for simultaneous optical stimulation and continuous monitoring of cortical activities

Darryl Narcisse, Sourajit Mustafi, Michael Carlson, Subrata Batabyal, Sanghoon Kim, Weldon Wright, Samarendra Mohanty*

Nanoscope Technologies LLC,

Bedford, TX 76022, USA.

* Correspondence should be addressed to S.M (smohanty@nanoscopetech.com)

The sequence of bMCOII is shown below:

MDYGGALSAVGRELLFVTNPVVVNGSVLVPEDQCYCAGWIESRGTNGAQTASNVLQWLA
AGFSILLLMFYAYQTWKSTCGWEEIYVCAIEMVKVILEFFFEFKNPSMLYLATGHRVQWLR
YAEWLLTCPVILIHLSNLTGLSNDYSRRTMGLLVSDIGTIVWGATSAMATGYVKVIFFCLGL
CYGANTFFHAAKAYIEGYHTVPKGRCRQVVTGMAWLFFVSWGMFPILFILGPEGFGVLSVY
GSTVGHTIIDLMSKNCWGLLGHYLRVLIHEHILIHGDIRKTTKLNIGGTEIEVETLVEDEAEAG
AMAELISSATRSLFAAGGINPWPNPYHHEDMGCGGMTPTGECFSTEWWCDPSYGLSDAGY
GYCFVEATGGYLVVGVEKKQAWLHSRGTPGEKIGAQVCQWIAFSIAIALLTFYGFSAWKAT
CGWEEVYVCCVEVLFVTLEIFKEFSSPATVYLSTGNHAYCLRYFEWLLSCPVILIRLSNLSGL
KNDYSKRTMGLIVSCVGMIVFGMAAGLATDWLKWLLYIVSCIYGGYMYFQAAKCYVEAN
HSVPKGHCRMVVKLMAYAYFASWGSYPILWAVGPEGLLKLSPYANSIGHSICDIIAKEFWTF
LAHHLRIKIHEHILIHGDIRKTTKMEIGGEEVEVEEFVEEEDEDTVVSKGEEDNMVSKGEEDN
MASLPATHELHIFGSINGVDFDMVGQGTGNPNDGYEELNLKSTKGDLQFSPWILVPHIGYGF
HQYLPYPDGMSPFQAAMVDGSGYQVHRTMQFEDGASLTVNYRYTYEGSHIKGEAQVKGT
GFPADGPVMTNSLTAADWCRSKKTYPNDKTIISTFKWSYTTGNGKRYRSTARTTYTFAKPM
AANYLKNQPMYVFRKTELKHSKTELNFKEWQKAFTGFEDFVGDWRQTAGYNLDQVLEQG
GVSSLFQNLGVSVTPIQRIVLSGENGLKIDIHVIIPYEGLSGDQMGQIEKIFKVVYPVDDHHFK
VILHYGTLVIDGVTPNMIDYFGRPYEGIAVFDGKKITVTGTLWNGNKIIDERLINPDGSLLFRV
TINGVTGWRLCERILA

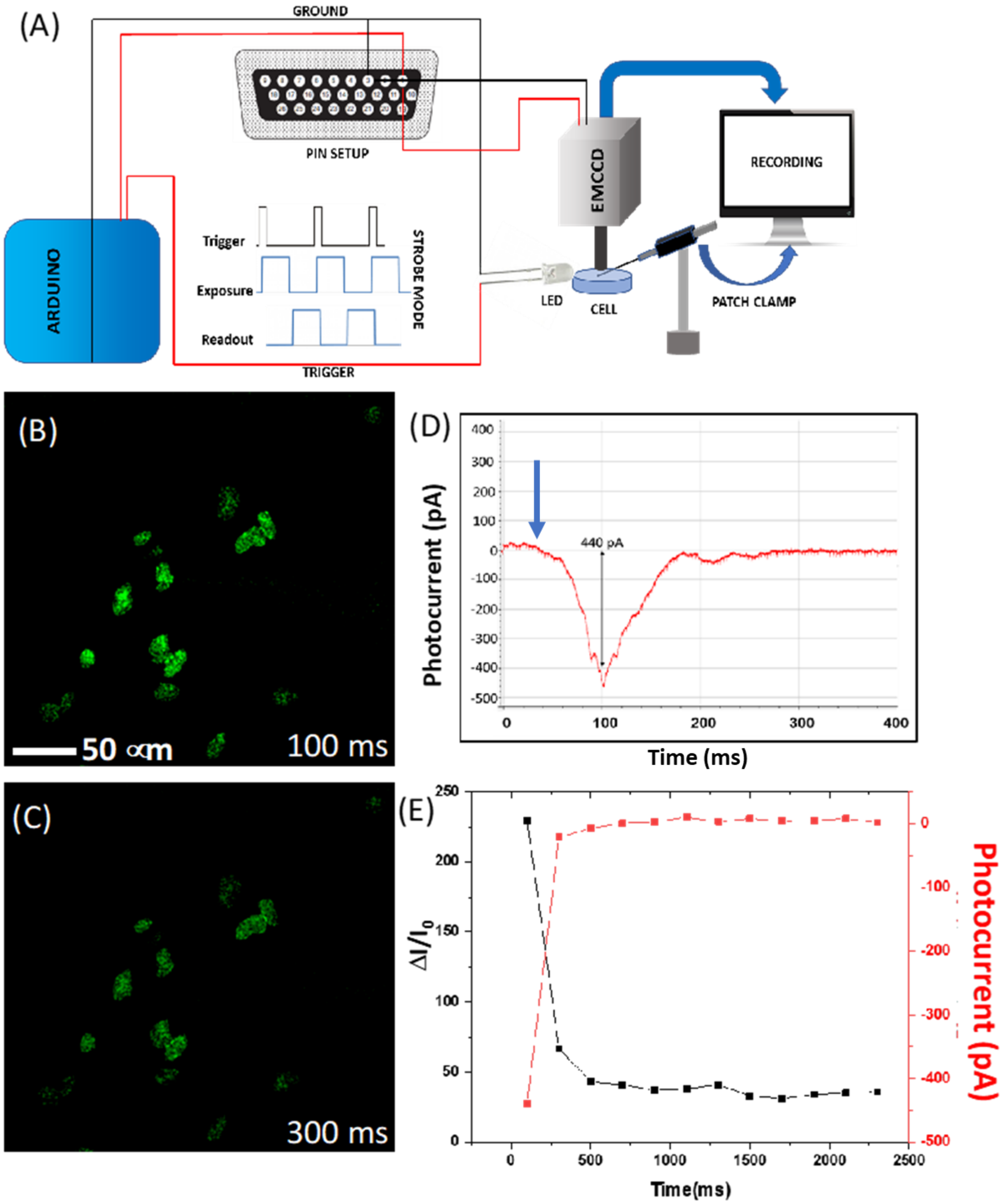

Supplementary Figure 1

Supplementary Figure 1. Correlation of Ca^{2+}-bioluminescence and inward photocurrent in bMCOII sensitized cells upon light stimulation. (A) Schematic diagram for simultaneous optical stimulation and recording of Ca^{2+}-bioluminescence along with patch-clamp electrophysiology. Time-lapse Ca^{2+}-bioluminescence from HEK293 cells at (B) 100 ms and (C) 300 ms after light stimulation. (D) Inward photocurrent recorded by patch-clamp in bMCOII expressing HEK293 cell stimulated by light (15 μW/mm^2). (E) Factional change in Ca^{2+}-bioluminescence and corresponding change in membrane current as a function of time.

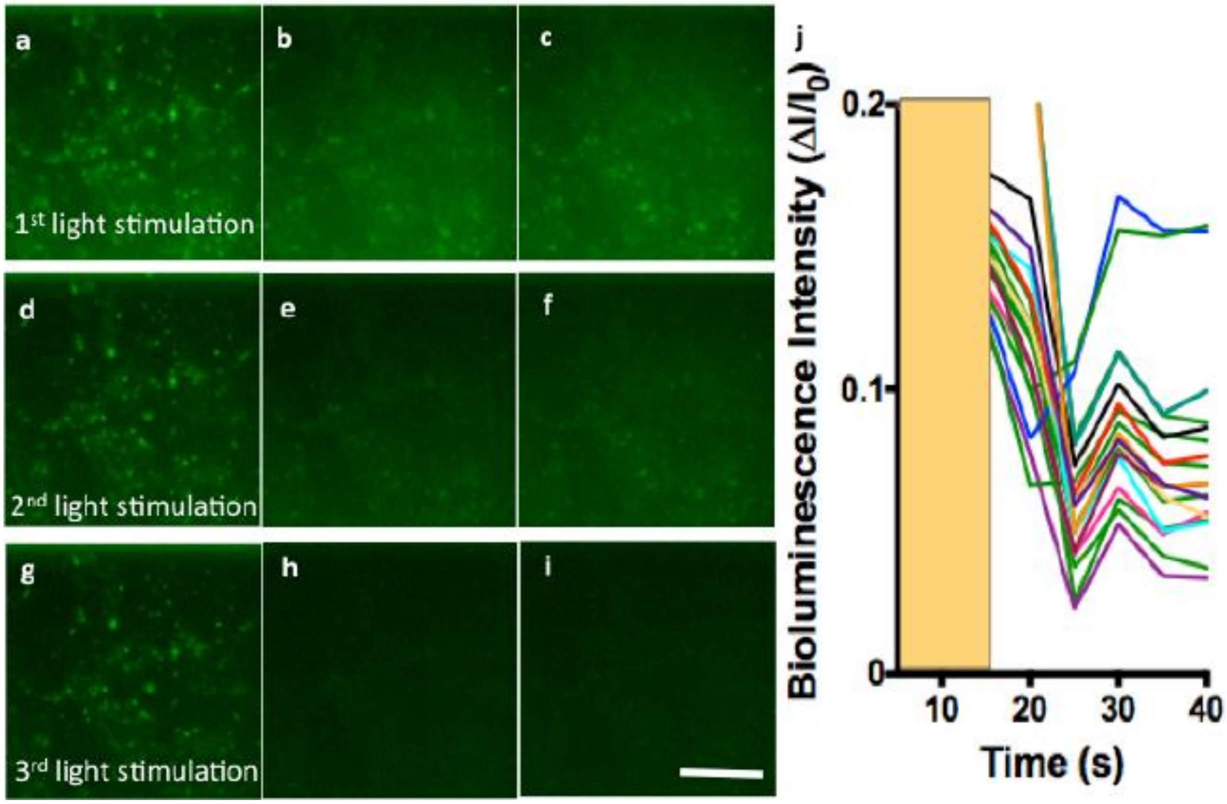

Supplementary Figure 2

Supplementary Figure 2. Light stimulated activities in HEK cells expressing both GeNL-Ca^{2+} and MCO-II monitored by bioluminescence. Bioluminescence mapping at 1, 10 and 15 seconds after 1st (a-c), 2nd (d-f) and 3rd (g-i) light stimulation. (j) Kinetics of bioluminescence in different cells in response to light stimulation (yellow bar, intensity: 20 μW/mm^2). Scale bar: 100 μm.

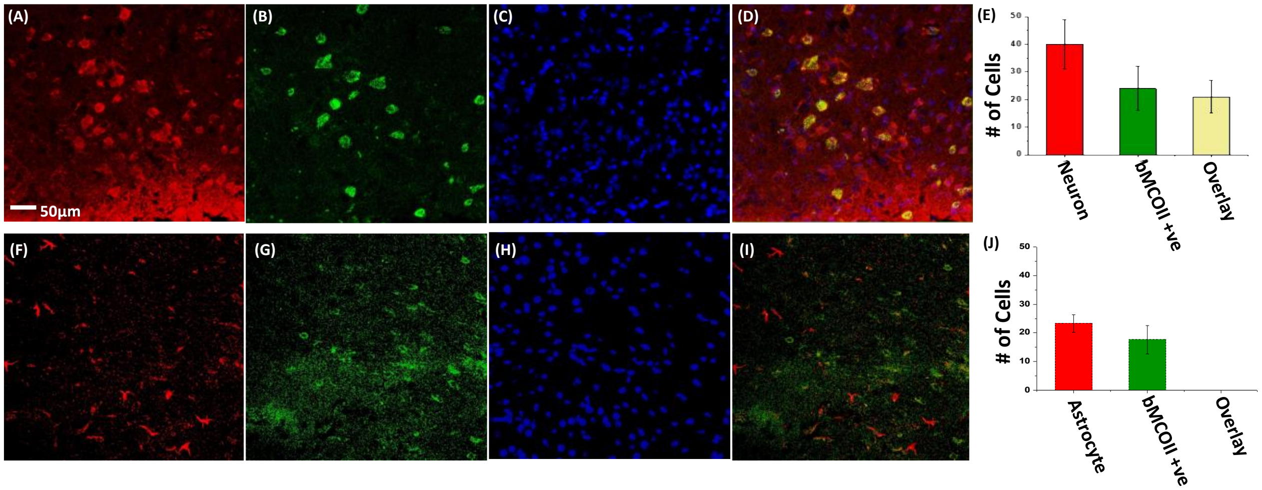

Supplementary Figure 3

Supplementary Figure 3. AAV2/5 based delivery of bMCOII is specific to cortical neurons. (A) ß3 tubulin immunofluorescence of neurons in a bMCOII-transduced cortical slice. (B) Intrinsic fluorescence of bMCOII, (C) DAPI staining of nuclei and (D) overlay of panels A, B and C showing co-localization. (E) Quantification of number of neurons expressing bMCOII. (F) GFAP immunostained cortical slice showing astrocytes. (G) Intrinsic fluorescence of bMCOII, (H) DAPI staining of nuclei and (I) overlay of panels F, G, and H showing no expression of bMCOII in astrocytes. (J) Quantification of number of astrocytes expressing bMCOII. The expression was quantified in 3 different 500 µm x500 µm regions of 20 µm brain sections. N=3. Av. ± SD. Scale Bar 50 µm.

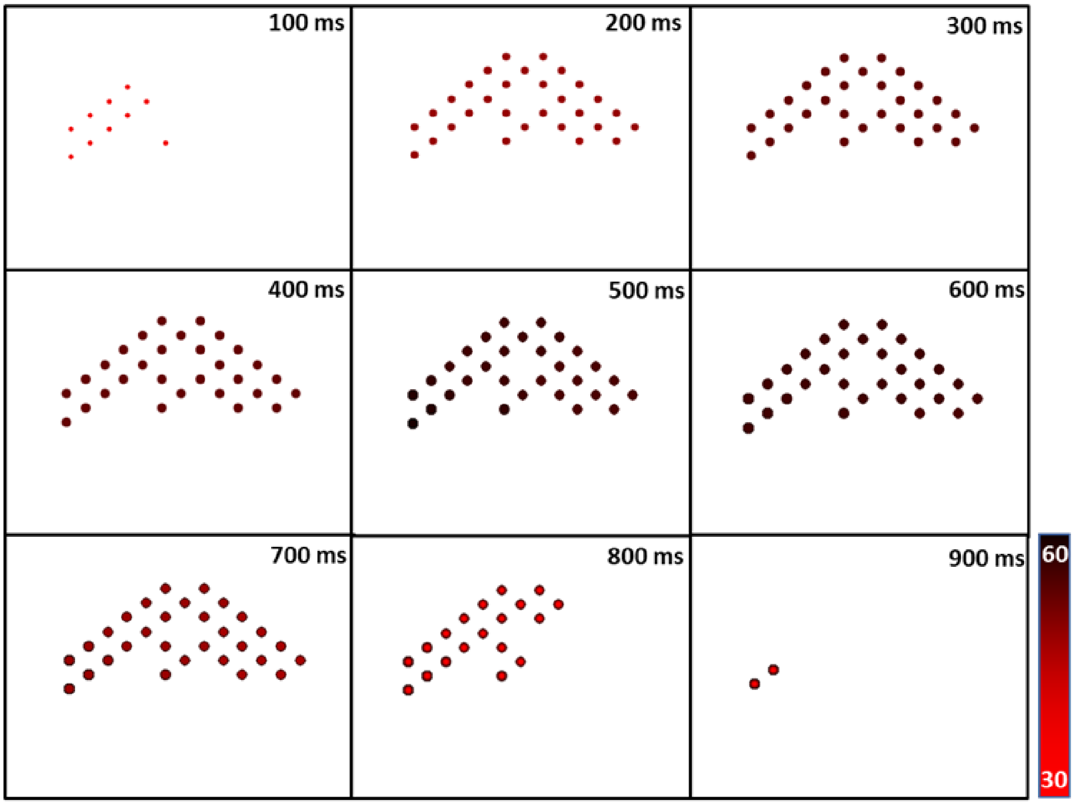

Supplementary Figure 4

Supplementary Figure 4. Time evolution of MEA signal in bMCOII sensitized cortical slices upon light stimulation. The radius of the circle represents number of voltage spikes and hot color represents average intensity of signal at each MEA electrode (diameter: 50µm, inter-electrode spacing: 200 µm). The color bar represents average extracellular potential in µV.

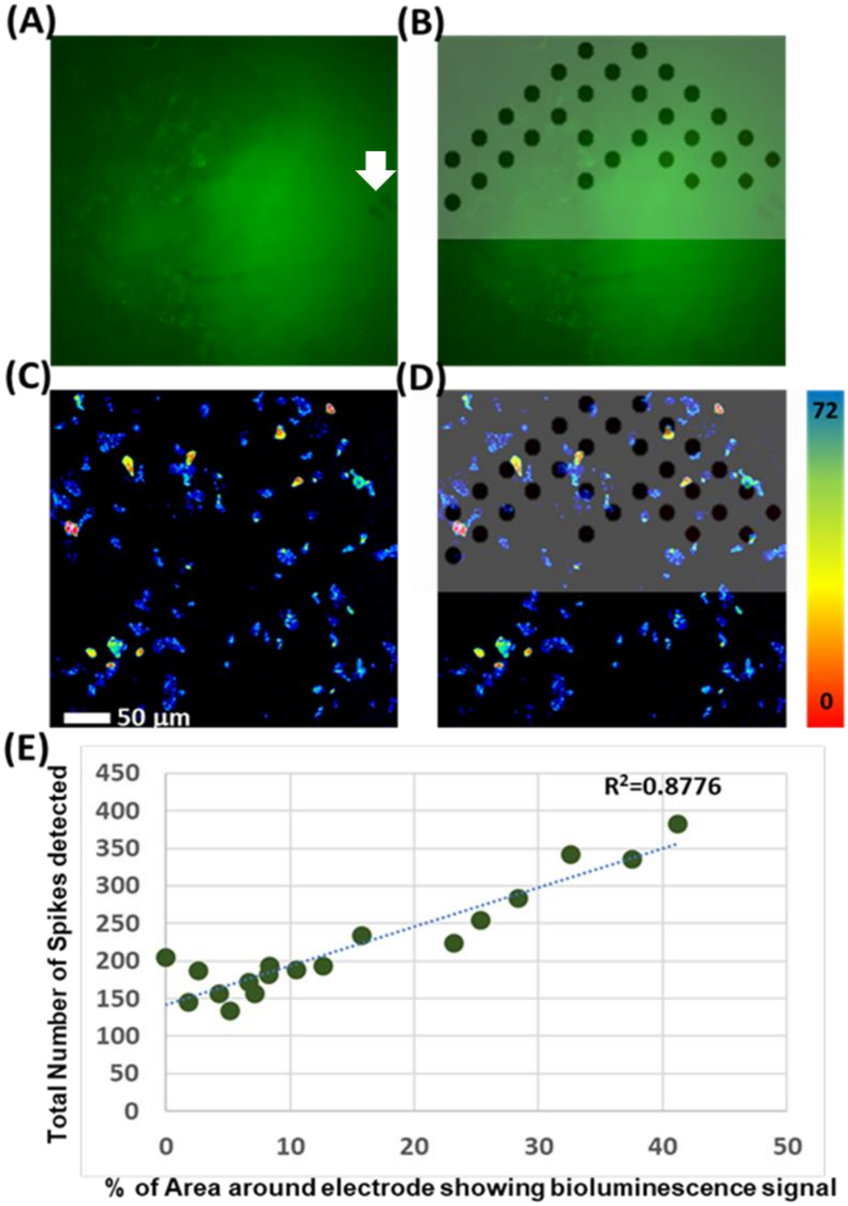

Supplementary Figure 5

Supplementary Figure 5. Spatial correlation of light induced Ca^{2+} bioluminescence signal and extracellular membrane potential in bMCOII sensitized cortical slice. (A) Epi-fluorescence image from cortical slice (Excitation: 490 nm, Emission: 540 nm) from the visual cortex transfected with 6 µl of 1.23×10^{13} GC/ml of AAV 2/5 carried bMCOII, 6-8 weeks prior to the recording. Arrow points to a landmark on the MEA plate. (B) MEA channels (that recorded light-stimulated neuronal spikes), as black circles overlaid on the fluorescence image. (C) Time-integrated Ca^{2+}-Bioluminescence signal from the cortical slice observed upon stimulation by blue light (470 ± 25 nm). (D) Registration of the MEA channels (that recorded light-stimulated neuronal spikes) with time-integrated Ca^{2+}-Bioluminescence signal. Pseudo color scale represents order of appearance after light stimulation. (E) Correlation with % of area around the electrode (diameter: 100 µm) showing Ca^{2+}-bioluminescence activity vs. total number of electrical spikes.

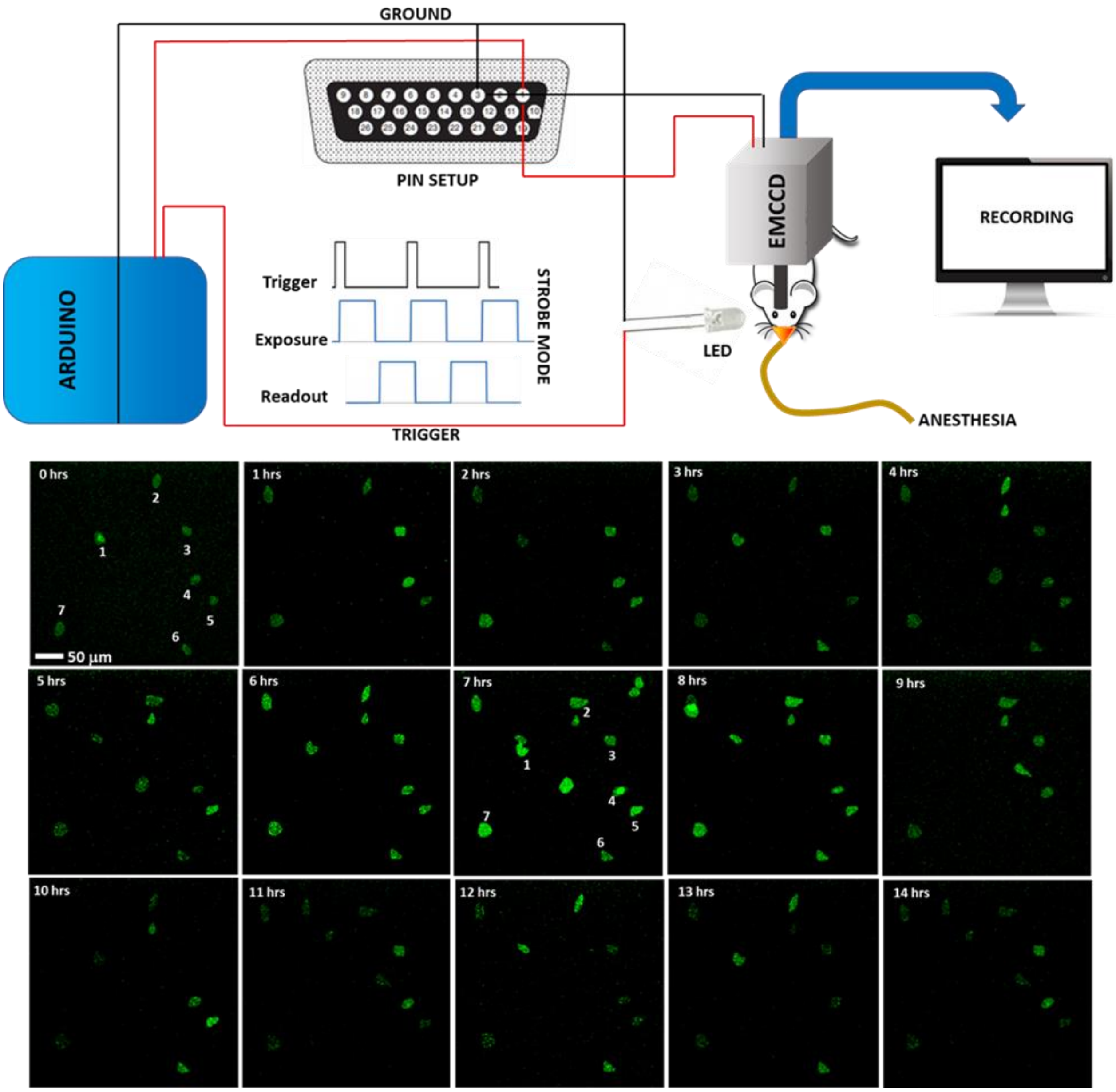

Supplementary Figure 6

Supplementary Figure 6. *In-vivo* visually evoked Ca^{2+}-bioluminescence activities in bMCOII transduced visual cortex of mice. Top: Schematic diagram of the in-vivo set up for stimulation-synchronized recording of visually evoked Ca^{2+}-Bioluminescence cortical activities. Wire diagram between Arduino UNO controller, LED source, and EMCCD camera. The Arduino is used for dead time synchronization of EMCCD and LED light stimulation, so that camera recording occurs when light stimulation is off. Bottom: Ca^{2+}-bioluminescence signal after first light stimulation at each time point during 0-14 hrs. duration. Peak Ca^{2+}-bioluminescence activities observed around 7 hrs. of anesthesia. N=3 mice.

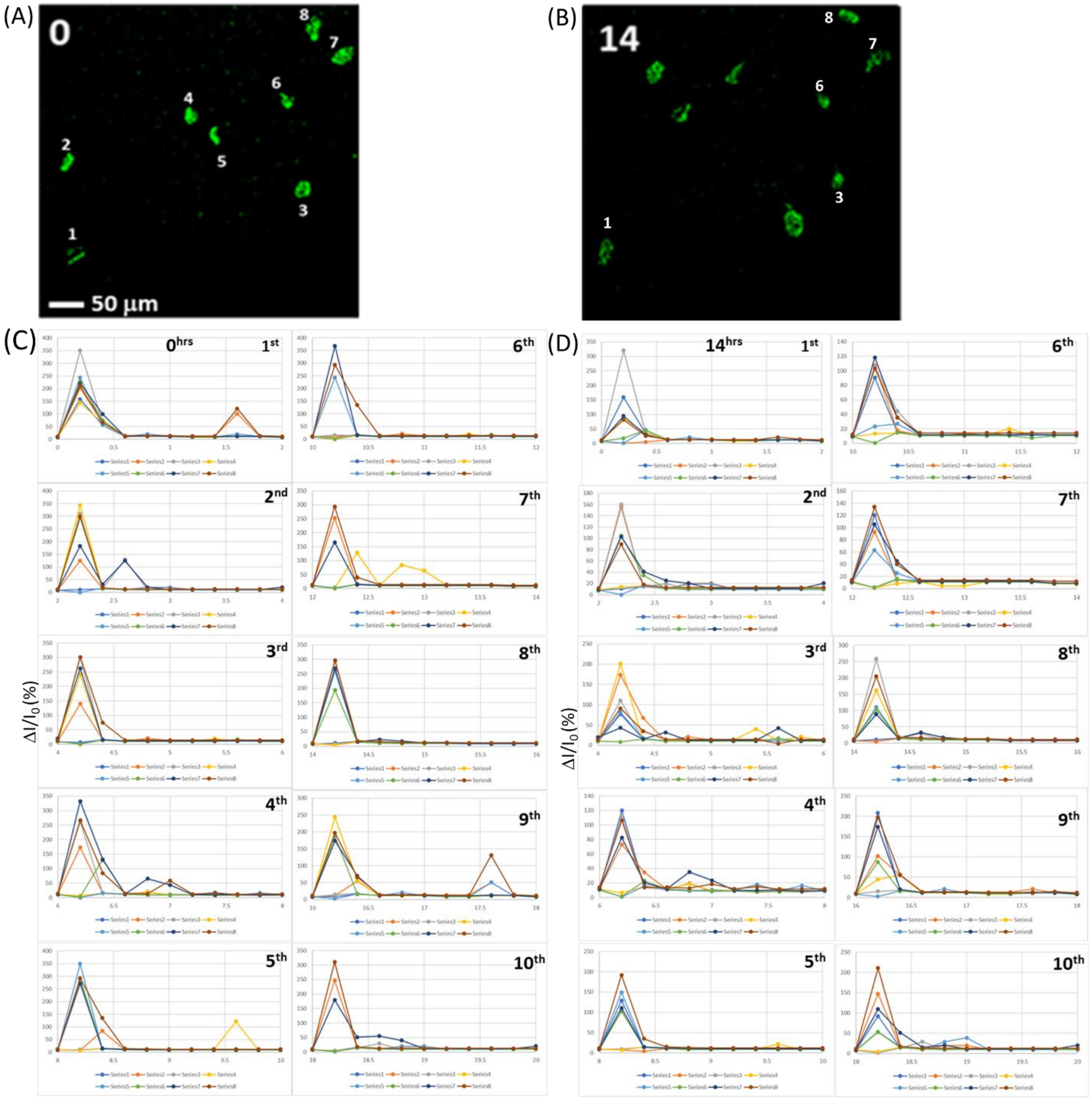

Supplementary Figure 7

Supplementary Figure 7. Fractional enhancement of Ca^{2+}-Bioluminescence intensity from cortical neurons of bMCOII transduced visual cortex and assignment of active neurons. Ca^{2+}-Bioluminescence image of visual cortex after 1st visual stimulation at (A) 0th and (B) 14th hour of continuous monitoring. The assignments of 8 active neurons are marked in the images. Plots of percentage change in Ca^{2+}-Bioluminescence intensity of 8 neurons (plotted in different colors, recorded with camera frame rate of 5 Hz) for 10 successive visual stimulations (10 ms pulse, 10 µW/mm^2, 0.5 Hz) at (C) 0th and (D) 14th hour.

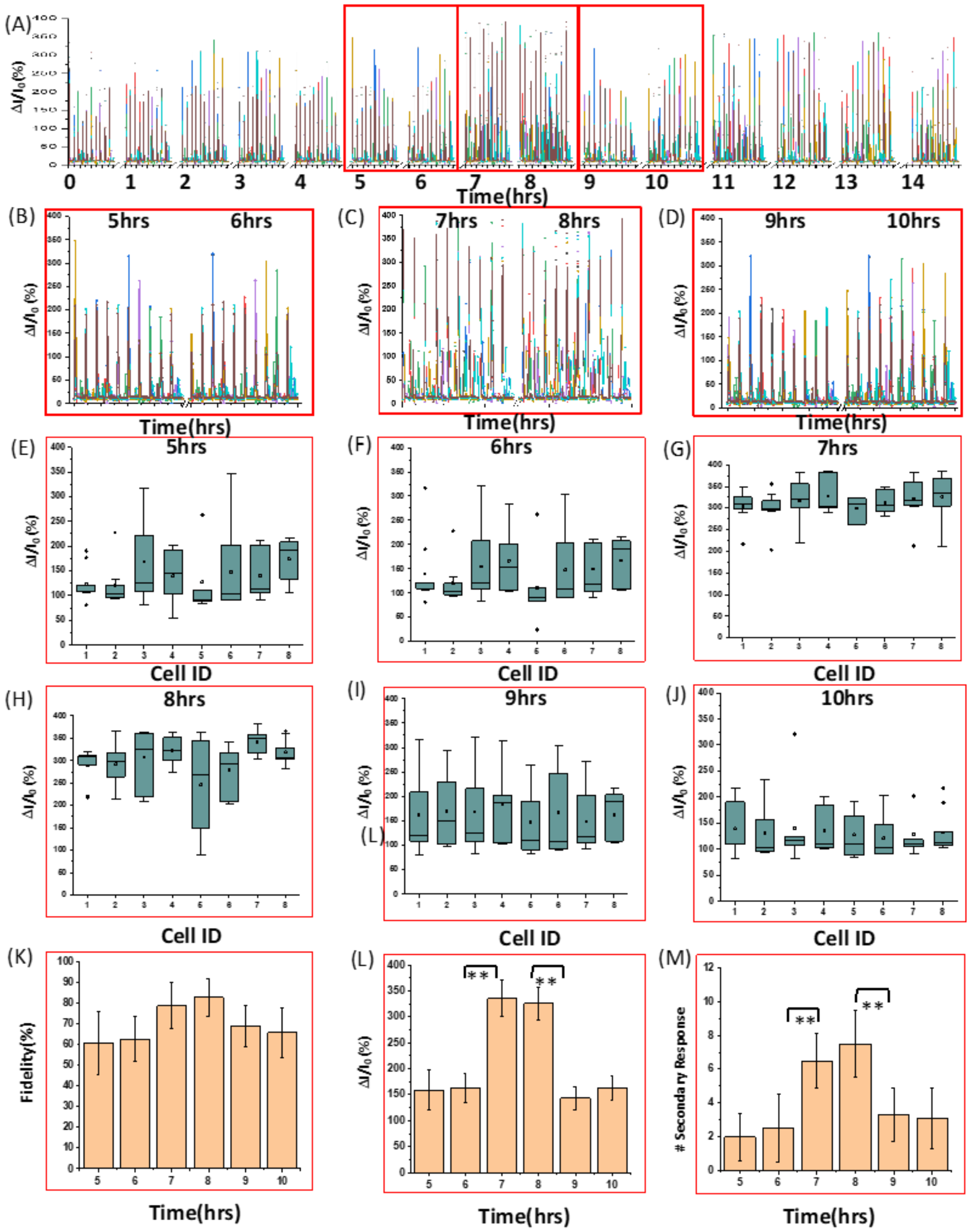

Supplementary Figure 8

Supplementary Figure 8. Continuous monitoring of Ca^{2+}-Bioluminescence signal in bMCOII transduced visual cortex upon low intensity light stimulation. (A) Continuous plot of fractional increase in Ca^{2+} bioluminescence signal for the 8 assigned active neurons for 14-hour period. (B-D) Ca^{2+}-bioluminescence changes for 5-6th, 7-8th and 9-10th hours, respectively. (E-J) Box plots showing average bioluminescence responses in 5th, 7th, 9th, 6th, 8^{th,} and 10th hours respectively for each of the 8 assigned neurons. (K) Fidelity, (L) Fractional increase in bioluminescence signal, and (M) number of secondary responses. * $p < 0.05$ and ** $p < 0.01$. Pulse width for light stimulation is 30 ms and intensity is 13 μW/mm^2.

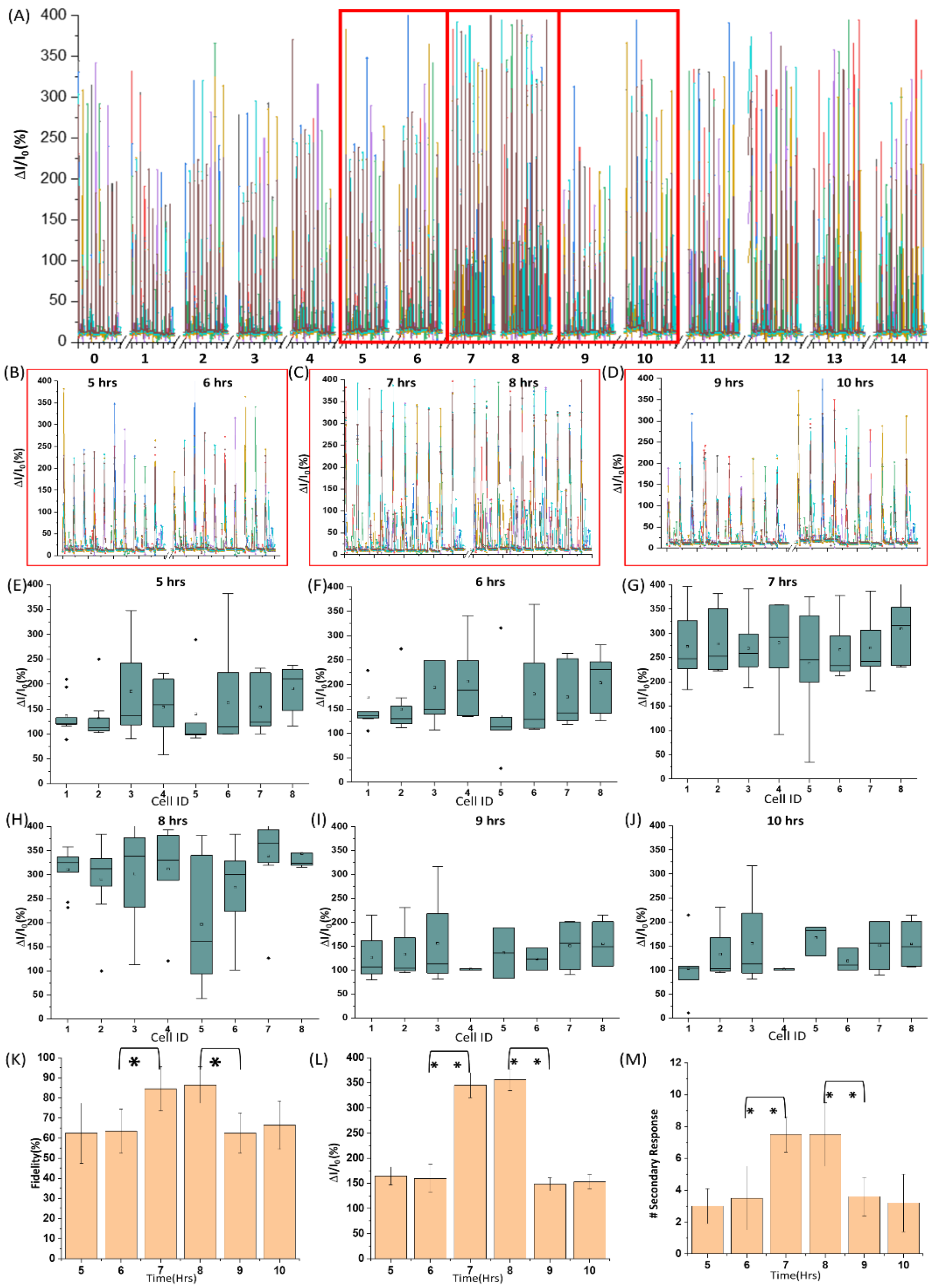

Supplementary Figure 9

Supplementary Figure 9: Continuous monitoring of Ca^{2+}-Bioluminescence signal in bMCOII expressing visual cortex upon high intensity visual stimulation. (A) Continuous plot of fractional increase in Ca^{2+} bioluminescence signal for the 8 assigned active neurons for 14-hour period. (B-D) Ca^{2+}-bioluminescence changes for 5-6th, 7-8th and 9-10t^{h} hours, respectively. (E-J) Box plots showing average bioluminescence responses in 5th, 7th, 9th, 6th, 8^{th,} and 10th hours respectively for each of the 8 assigned neurons. (K) Fidelity, (L) Fractional increase in bioluminescence signal, and (M) number of secondary responses. * $p < 0.05$ and **$p < 0.01$. Pulse width for light stimulation is 30 ms and intensity is 22 μW/mm^2.

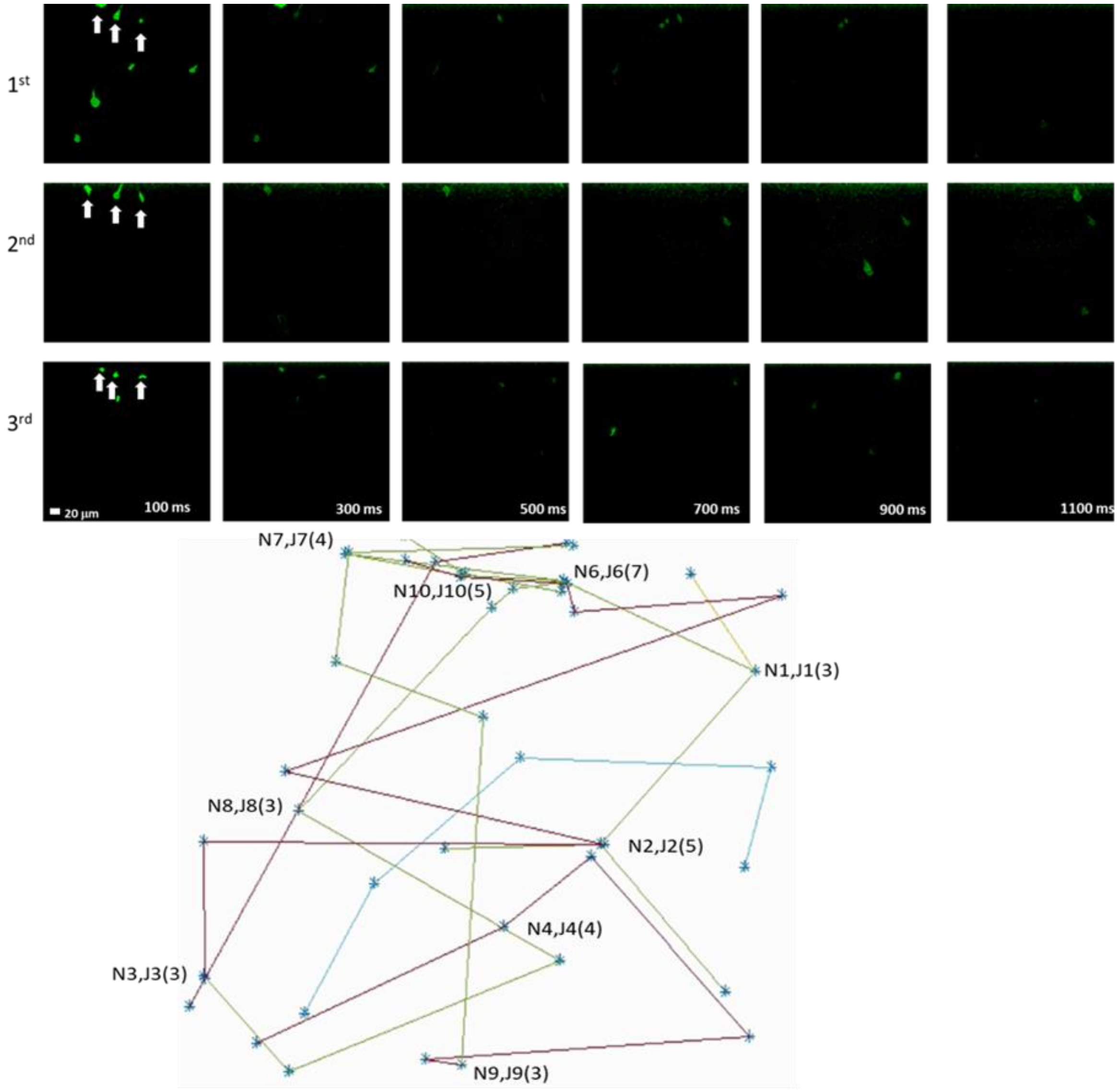

Supplementary Figure 10

Supplementary Figure 10. Characterization of neural activity architecture in visual cortex expressing bMCOII upon light stimulation. Top: Time course of Ca^{2+}-bioluminescence signal from visual cortex of wild type mice upon illumination of eye with white light (intensity: 12 µW/mm^2, pulse width: 10 ms). The camera exposure was 100 ms. Three successive time-lapse images upon light stimulation have been shown. White arrows show repeating signals from cortical neurons after visual stimulus. Bottom: Artificial Intelligence assisted neural activity network pattern in cortex following light stimulation. The nodes and junctions are evaluated using convolution neural network (CNN). The neural activation parameter is defined as the convolution of bioluminescence intensity and activity architecture.

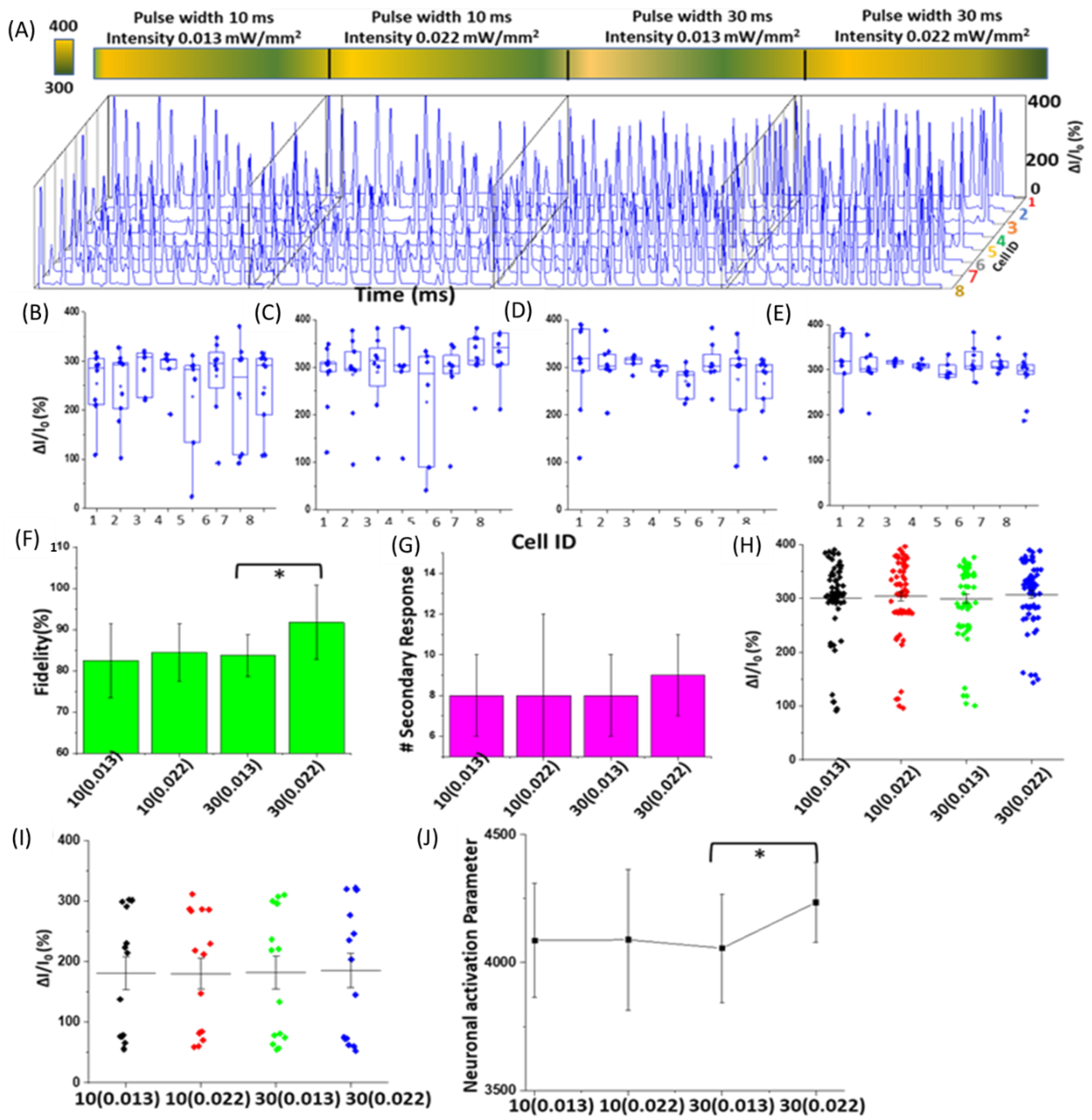

Supplementary Figure 11

Supplementary Figure 11. Comparison of *in-vivo* Ca^{2+}-bioluminescence activities and neural activation parameter in visual cortex expressing bMCOII upon light stimulation at different intensities and pulse widths. (A) The 3D plot of fractional change in bioluminescence intensity from 8 different active neurons for various stimulation parameters (intensity and pulse width, depicted in pseudo color) at 8th hour of recording. (B-E) Boxplot showing fractional change in Ca^{2+}-bioluminescence intensity for various stimulation parameters. (F) Fidelity, (G) Number of secondary responses. Scatted plot of fractional change in bioluminescence signal for (H) primary response, and (I) secondary response, for various stimulation parameters. (J) Calculated neuronal activation parameter for various stimulation parameters. The X-axis represents various stimulation parameters: Pulse width in ms (Intensity in mW/mm^2). * $p < 0.05$.

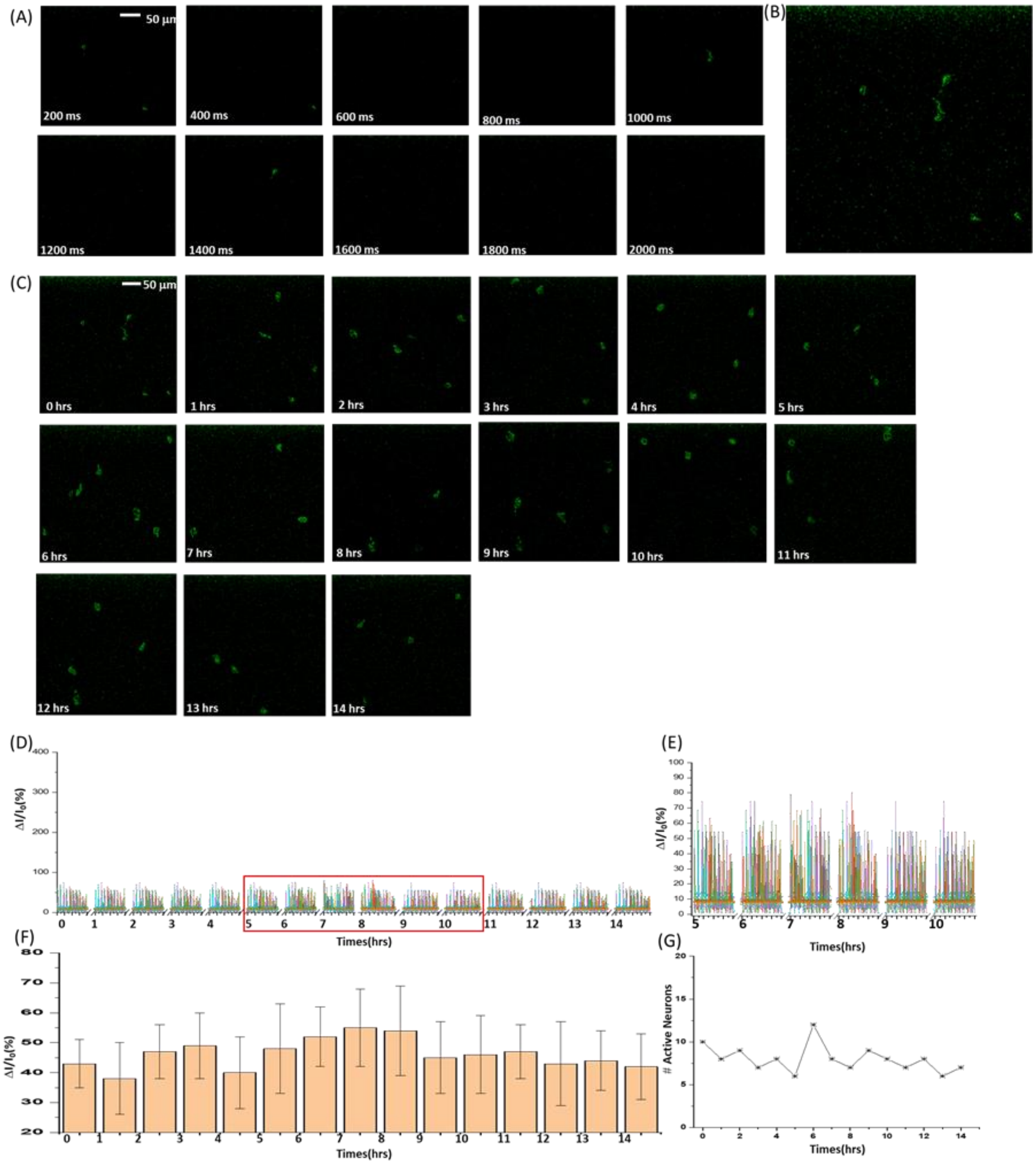

Supplementary Figure 12

Supplementary Figure 12**. *In-vivo* baseline Ca^{2+}-bioluminescence activities in bMCOII transduced visual cortex of mice.** (A) Time-lapse images of Ca^{2+}-bioluminescence activities in visual cortex in wild type mice at 0 hrs. (B) Time-integrated (over 2 sec period) Ca^{2+}-bioluminescence signal at 0 hrs. (C) Time-integrated (over 2 sec) Ca^{2+}-bioluminescence signal at every hour during 0-14 hrs. duration. (D) Continuous plot of fractional increase in Ca^{2+}-bioluminescence signal for the active neurons for 14-hour period. (E) Zoomed image of Ca^{2+}-bioluminescence changes in 5-10 hours period. (F) Average Ca^{2+}-bioluminescence signal and (G) Number of active neurons for 14 hours period. The highest number of active neurons were observed at 6th hour of recording (10th ZT hour).

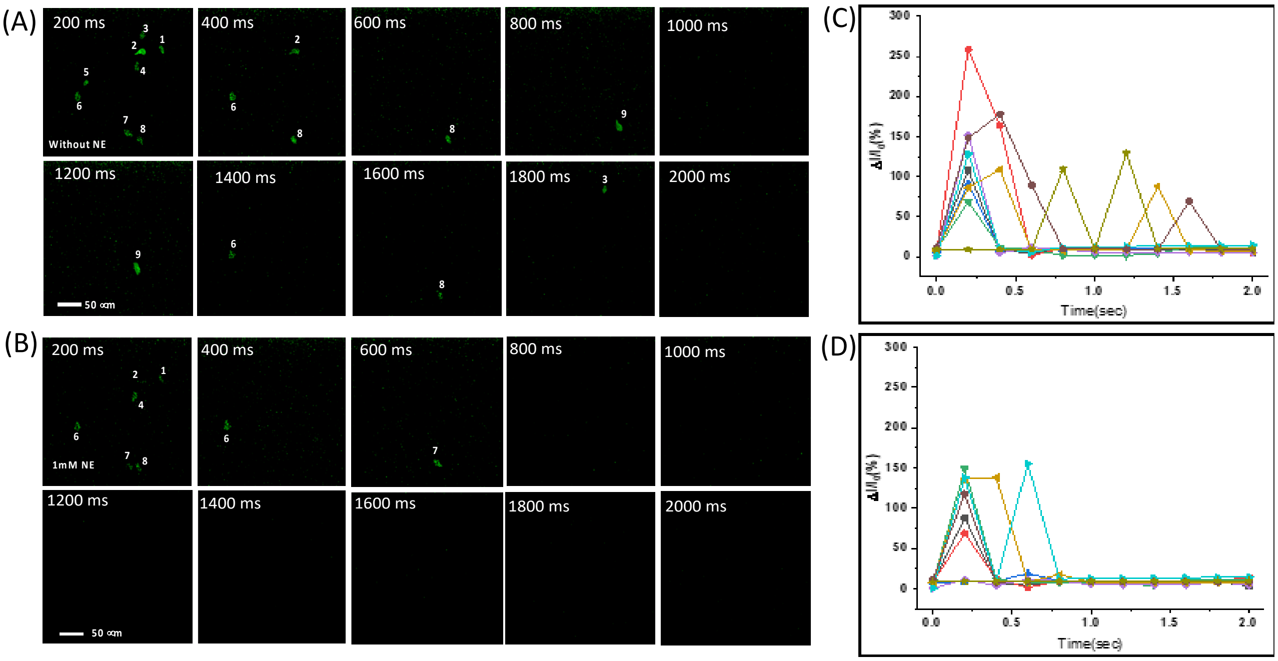

Supplementary Figure 13

Supplementary Figure 13. Characterization of neuron-astrocyte communication in visual cortex expressing bMCOII upon visual stimulation. Time-lapse images of Ca^{2+}-bioluminescence activities in visual cortex of wild type mice upon illumination of eye with white light (intensity: 12 µW/mm^2, pulse width: 10 ms) (A) Before and (B) after application of 1 mM Norepinephrine (NE). Plots of fractional change in Ca^{2+}-bioluminescence intensity of assigned neurons (plotted in different colors, recorded with camera frame rate of 5 Hz) in one visual stimulation period (C) Before and (D) after application of 1 mM NE. Addition of 1 mM NE completely suppresses the secondary responses.
